# Kraft Cooking Kinetics of Poplar Wood Chips Pretreated via a Mechano-Enzymatic Method for Further Nanocellulose Production

**DOI:** 10.3390/molecules31193384

**Published:** 2026-09-23

**Authors:** Arthur Valencony, Sandra Tapin-Lingua, Seyedeh Hadis Hashemi, Gerard Mortha

**Affiliations:** 1 Institut Technologique FCBA, Domaine Universitaire, CS 90251, CEDEX 9, 38044 Grenoble, France; sandra.tapin-lingua@fcba.fr; 2 Laboratory of Process Engineering for Biorefinery, Bio-Based Materials and Functional Printing (LGP2), University Grenoble Alpes (UGA), 38000 Grenoble, France; gerard.mortha@grenoble-inp.fr; 3 Département de Biologie Moléculaire et Cellulaire, UFR Chimie et Biologie, Domaine Universitaire Campus Saint Martin d’Hères, University Grenoble Alpes (UGA), 621 avenue Centrale, 38400 Saint-Martin-d’Hères, France; seyedeh-hadis.hashemi@etu.univ-grenoble-alpes.fr

**Keywords:** kraft cooking, kinetics, mechanical pretreatment, xylanase pretreatment, poplar chips

## Abstract

Kraft cooking kinetics of poplar wood (*Populus deltoides* and *Populus nigra*) were investigated using chips of controlled size, and various enzymatic/mechanical pretreatments of the chips were attempted to observe the effects on pulp quality. Batch kraft cooks of small amounts of untreated wood chips were carried out in mini-autoclaves at a range of 140–165 °C. Delignification rate constants and apparent activation energy during the bulk phase were determined. Various mechanical pretreatments, using a modular screw device (MSD) with or without xylanase impregnation prior to the cooks, were also investigated. Cooks were monitored through pulp yield, kappa number, fiber morphology, viscosity-average degree of polymerization (DPv), and residual effective alkali (REA). The results showed a variable, temperature-dependent activation energy of about 100 kJ/mol for the bulk delignification with a non-linear Arrhenius behavior suggesting a shift from reaction-controlled to diffusion-controlled kinetics as the temperature increased. The effectiveness of xylanase impregnation was limited due to chip thickness that limited enzyme penetration. MSD and MSD-Xylanase pretreatments significantly reduced fiber length (from 871 µm to 845 µm and 784 µm, respectively) and increased fines content (from 20.3% to 26.3% and 29.1%, respectively), indicating degraded fiber quality after the cooks of pretreated chips. Variability in pulp yield and REA was too high to reach statistical significance. Finally, it was shown that neither of these pretreatments could improve pulp quality in terms of fiber morphology and pulp strength properties, but this study opens the way toward the utilization of pretreatments to improve pulp fibrillation for nanocellulose production.

## 1. Introduction

Today, the kraft process is the most widely used in the world for the production of wood paper pulp, board, and packaging [1]. Understanding the mechanism and kinetics of delignification is essential for improving pulp yield and optimizing process parameters (temperature, time, reagent loads and concentrations, alkali consumption). Several kinetic models have been proposed for different wood species (softwood and hardwood) to describe cooking kinetics. In the classical approach, delignification is divided in various phases (generally three) [2,3,4,5], as has been illustrated in the Gustafson cooking model [6]: the initial phase, where a 15–40% range of lignin is eliminated, accompanied by strong extractives and hemicelluloses dissolution; the bulk phase, where most of the lignin in the secondary wall and middle lamella is dissolved and cellulose is partly depolymerized; and the residual phase, where delignification becomes very slow, some lignin recondensation on fibers takes place, and polysaccharides are damaged [4,7,8]. Another kinetic description was developed in Purdue University by Smith [9], which assumed that lignin in wood was composed of several coexisting fractions with different reactivities, each one with its own kinetic rate and activation energy. Such a description introduces a particular kinetic complexity coming from the reactivity distribution.

Apart from conventional paper/board applications requiring particular pulp and fiber specifications (chemical composition, physical strength and other properties), kraft fibers have also been used as a substrate for nanocellulose production [10,11,12,13]. In this case, keeping a high production yield is important because of the many process steps involved. One possible improvement approach, compared to classical kraft cooks, is to pretreat the biomass before the cooks. Pretreatments before the cooks can be divided into three categories: (1) mechanical treatments such as extrusion, defibrillation, and steam explosion, which act on biomass through cutting, shearing, compressing, tearing, or breaking [14]; (2) chemical pretreatments using mild acids or bases, organic solvents, or DESs (Deep Eutectic Solvents) [15]; and (3) biological pretreatments using enzymes, such as multicopper oxidase (MCO) laccase that oxidizes lignin [16,17,18], xylanase, which modifies wood xylans [19], or residual xylans after cooking, to enhance delignification and defibrillation [20]. In the present study, mechanical (shearing and compression) and enzymatic (xylanase) pretreatments were investigated, individually and in combination. However, it is known that such pretreatments can significantly affect fiber quality in pulp production even though they may be useful for biomass deconstruction toward nanomaterials production.

Mechanical pretreatments can be used separately and generally require multiple steps, bulky equipment, and are energy-consuming devices [14]. Combining a mechanical pretreatment with theaction of enzymes in a “mechano-enzymatic” pretreatment might simplify the entire process, save mechanical energy, and potentially improve kraft cooking yield and kinetics.

However, the synergy between action of enzymes and the severity required for mechanical action is not straightforward. Mechanical pretreatments break the wood and cell wall macrostructure, thereby increasing the accessible internal area and creating cracks at the interfaces of the different layers of the fiber wall (between the primary and secondary wall, and between the middle lamella and the S1 layer). Such a mechanical action can improve enzyme penetration and mass transfer in the cell wall [21]. However, simultaneously, an enzymatic action on wood polymers prior to the cooks may increase water uptake into the cell wall through partial hemicellulose depolymerization, while the resulting solubilized xylan fragments can redeposit and precipitate onto cellulose fibers [22]. This xylan redeposition has been reported to hinder lignin removal by limiting reagent diffusion across the cell wall during subsequent cooking, thereby acting as a disbenefit for delignification despite the initial hydrolytic action of the enzyme [22].

In our study, poplar wood was chosen since it is a low-density/high-porosity wood used as raw material for the production of wooden crates. Indeed, poplar wood represents an attractive raw material for pulp production owing to its relatively open wood structure, which can facilitate liquor penetration and mass transfer within wood chips. It is also a reject from the plywood industry that can be used for chemical pulping [23,24]. For cooking, it is a rather interesting wood because of its low density, which gives it great diffusion capabilities, a property that can enhance liquid and chemicals penetration and molecular diffusion in the cell wall of chips. Such structural characteristics may also be advantageous for enzymatic pretreatment by increasing the accessibility of the lignocellulosic matrix to hydrolytic enzymes. Moreover, poplar is interesting for its rapid growth and rather high content in polysaccharides, compared to some other hardwoods (fewer extractives and lignin) [25]. It is generally cooked more easily than other hardwoods and does not require a high temperature during the bulk phase. Thus, poplar appears to be a relevant substrate for investigating enzyme-assisted kraft pulping, where improved enzyme accessibility can potentially enhance xylan hydrolysis and facilitate subsequent delignification.

Endoxylanases are enzymes that will randomly cleave β-1,4 bonds in xylans, which helps improve the so-called “biobleaching” of lignocellulosic fibers. Indeed, it is well-known that xylan degradation improves delignification [26,27] and chromophore elimination. Chromophores are responsible for the brown color of pulp and its brightness reversion under heat and light exposure [28]. Xylanases should therefore reduce chemicals consumption during delignification and bleaching, but also improve fiber fibrillation and decrease refining time [29]. Xylanase specifically targets xylans, which are involved in lignincarbohydrate complexes (LCCs), which can limit reagent access to lignin during kraft cooks: it is estimated that 90% of residual lignin is covalently linked to hemicelluloses [27]. This makes it the most relevant enzyme for this study’s objective, despite its low overall activity on intact wood. Laccases, although potentially more active on lignin, generally require a mediator system to act efficiently on native lignin; without one, their action remains largely superficial [30].

However, in general, enzymatic treatments applied on raw wood chips are considered ineffective due to the steric exclusion of enzymes from the cell wall polymeric structure (the size between aggregated polysaccharide chains being lower than 2 nm, whereas the hydrodynamic radius of enzymes are larger than 4 nm) [31,32]. Enzyme diffusion is recognized to be inefficient in the relatively closed and inaccessible structure of wood [33], although it can be a cost-effective solution to improve cooking efficiency and, possibly, pulp properties. In 2017, Walia et al. [20] noted that enzymatic pretreatment prior to kraft cooking has not been systematically evaluated across different cooking scales. More recently, Pandey et al. [34] combined mechanical pretreatment and xylanase impregnation of *Eucalyptus tereticornis* wood chips prior to kraft cooking, showing that this mechano-enzymatic approach could reduce the required cooking temperature and improve pulp strength properties, supporting the relevance of the approach investigated in the present study. However, as highlighted in a recent review on xylanase applications in the pulp and paper industry [35], most enzymatic treatments reported in the literature are still applied on already-pulped material for bleaching purposes, rather than on raw wood chips prior to cooking, leaving the kinetic impact of such pretreatments on kraft cooking itself largely unexplored.

This work evaluates the kraft cooking kinetics of controlled-size poplar wood chips. The bulk-phase delignification rate constants at different temperatures were determined and the activation energy was calculated. In addition, the impacts of xylanase impregnation and mechanical pretreatment on wood chips were studied with the objective not to demonstrate an important enhancement of delignification, but to quantify the deviation from an expected null effect. Therefore, the potential effect of mechanically opening the wood structure by a strong mechanical pretreatment (using a Modular Screw Device (MSD) apparatus) was evaluated. Finally, batch reactions at a larger scale were carried out in bigger autoclaves on industrial wood chips, and the trends observed during mini-autoclave cooks (being considered as a comparative screening tool) were analyzed and compared to cooking results in larger autoclaves by an ANOVA analysis. Observations and data analysis rely on measuring the pulping mass yield, the kappa number, the residual effective alkali (REA), and the DPv (viscosity-average degree of polymerization) of the different pulps produced. Fiber morphology characterizations were also added.

## 2. Results and Discussion

### 2.1. Results for Rectangular Wood Chips

The poplar wood chips were precisely cut into a parallelepiped geometry, with constant dimensions. Indeed, capillary penetration is well-controlled in this way because of the good alignment of fibers in the longitudinal direction, orthogonal to chip thickness (in the radial direction).

Kraft cooks were performed at temperatures ranging from 140 °C to 165 °C. For short cooking times, the kappa number could not be determined because of the high lignin content in the samples.

Results from the small autoclave reactions are presented in Figure 1A–C (the detailed data are available in Supplementary Materials file in Table S1).

The total pulp yield (unscreened pulp yield) during cooks is shown in Figure 1A. At 140 °C, the yield decreased very slowly, reflecting incomplete delignification and a limited dissolution of carbohydrates at the end of the cook. At 145 °C, the yield decreased more sharply from approximately 80 wt.% to 64 wt.% during the first hour and then stabilized at about 60 wt.%, indicating a gradual transition to the bulk phase. At 155 °C, a sharp yield decrease is observed between 30 and 150 min, consistent with bulk delignification, followed by yield stabilization during the residual phase. This temperature seems like a good compromise for a balance between efficient delignification and yield retention (polysaccharide preservation). In contrast, cooking at 165 °C resulted in a strong initial yield loss (~ 60 wt.%) followed by stabilization at about 55 wt.% pulp yield, the lowest value of the series. This indicates a fast chemical attack on lignin and strong polysaccharide degradation. Although delignification was efficient, fiber quality may have been compromised. Overall, it was found that, at this temperature range for poplar chips, the final yield presented a rather sharp decrease with increasing temperature, consistent with previous studies [36]. Final pulp yield values were 63.7 wt.% at 140 °C, 59.0 wt.% at 145 °C, 55.9 wt.% at 155 °C, and 55.7 wt.% at 165 °C. Typical values for industrial kraft process are around 50 wt.% up to 54 wt.% with aspen [37].

The Residual effective alkali (REA) profiles presented in Figure 1B are complementary to the yield observation. At 140 °C, REA decreased rather gradually (almost linearly) during the cook, reflecting the behavior of a prolonged initial phase and the beginning of the bulk phase without visible transition. At 145 °C, alkali consumption appeared non-linear, indicating the occurrence of the bulk and residual phases. At 155 °C, rapid initial alkali consumption was observed, consistent with the strong delignification reflected by the yield loss and kappa number evolutions presented in Figure 1C. At 165 °C, alkali consumption was the fastest of the series and was consistent with rapid delignification and increased yield loss due to polysaccharide degradation. Final REA values gradually decreased with temperature from 5.4 g.L^−1^ at 140 °C, 7.2 g.L^−1^ at 145 °C, 3.8 g.L^−1^ at 155 °C, and 2.0 g.L^−1^ at 165 °C.

The kappa number (KN) evolution during the high-temperature plateau (after 150 min of cooking) is shown in Figure 1C. At 140 °C, the KN was rather stable until 210 min, corresponding to the continuation of the initial phase; then, it decreased continuously but moderately during the bulk phase (KN of 59.2 at 300 min, and 56.6 at the end of the cook; no residual phase). Similarly at 145 °C, no residual phase was observed and a smooth transition between initial and bulk phases occurred.

At 165 °C, the KN decreased rapidly after 150 min (bulk phase) and it stabilized during the residual phase, ending at a KN range of 16–17. The same effect was observed at 155 °C, but the residual phase started later and ended at a KN range of 21–22. Final kappa values were 56.6 at 140 °C, 39.5 at 145 °C, 21.3 at 155 °C, and 15.9 at 165 °C.

During both kraft and soda cooking, it is generally accepted that the residual lignin content (L) in wood (initial wood basis) can be approximately calculated from the kappa number and pulp yield by the following relationship (although this relation does not account for the possible presence of hexenuronic acids):

Lignin content (g of lignin/ g of initial wood)=Kappa number × k × Yield/100 

Such a relationship generally holds for KN values below 80 (the beginning of the high-temperature plateau in our study). For hardwood kraft cooks, the proportionality constant k is generally estimated between 0.16 and 0.17 [5,38,39,40], and the value of k = 0.165 was used in this study. Since the delignification kinetic generally follows a first-order kinetic law with respect to the lignin content (L), the classical representation of (log_10_ (L) vs. plateau time) during the bulk phase is shown in Figure 2. As expected, the curve fit in Figure 2 is found to be rather linear.

Kinetic rate constant *k*-values at a given temperature can be derived from the slope of each curve. By applying the Arrhenius law, $k=k_{0}{\;e}^{\frac{E_{a}}{RT}}$, one obtains the apparent activation energy E_a_ from the classical Arrhenius curve represented in Figure 3:

$$ln\;\left(k\right)=\ln\left(k_{0}\right)-\;\frac{E_{a}}{RT}$$

By linear regression, one obtains $\frac{-E_{a}}{R}$ = 12,090 K, and then E_a_ = 100.5 kJ.mol^−1^ (taking R = 8.314 J.mol^−1^.K^−1^).

In 2009, Marta Margarido (PhD thesis) [5], working in similar cooking conditions and with the same cooking device, obtained a value of E_a_ = 131 kJ.mol^−1^ for poplar wood-chip cooking, rather close to the activation energy value given for the H-factor (134 kJ.mol^−1^). However, in Margarido’s study, different wood chips were used: thinner and with different morphologies (half-moon shape). Moreover, poplar wood did not originate from the same ground parcel, and the species was different. In our study, the lower average E_a_ value found for the global delignification process suggests that mass transfer (reagent diffusion inside the chip wall) and chemical reactions were simultaneously involved as limiting processes during delignification. Moreover, it can be seen in Figure 3 that the Arrhenius plot is not fully linear. The apparent change in slope suggests a transition from chemical reaction limitations at low temperatures to diffusion limitations at higher temperatures. This effect has been demonstrated on birch wood meal where a new delignification model has been proposed, based on lignin molecular weight and diffusivity of dissolved lignin fragments across the thickness of the cell wall [41]. They demonstrated that the global delignification rate is mostly limited by the diffusion of lignin fragments, whereas alkali diffusion and lignin fragmentation reactions have a lower impact on overall delignification kinetics.

Figure 4 shows some morphological characteristics of the obtained fibers after cooking, measured by the Morfi analyzer. At 140 °C, because of the incomplete delignification, fiber morphology and DPv could not be measured. The global trend is a decrease in fiber length, which correlates with kappa number evolution: higher delignification increases fiber separation and the creation of the smallest elements. The fines content slightly decreases but the difference between 155 °C and 165 °C does not seem relevant.

The effects of temperature on fiber morphology at the end of the cook are typically illustrated by the optical microscope images shown in Figure 5A–D. Microscopic observations at 140 °C and 145 °C reveal a predominance of poorly separated fibers or shives. The lignified fibers—as evidenced by the staining obtained with Herzberg’s reagent, where some areas remain brown rather than blue—are assembled into fiber bundles. On the contrary, at a 155 °C cook temperature, fibers are well-individualized. These fiber bundles obtained at lower temperatures are also clearly visible under SEM (Figure 5E–H). The SEM images reveal good separation without degradation of the fiber wall for cooking under more intense conditions (155 °C and 165 °C), which exhibits a smooth structure.

Figure 6 shows the DPv of the final samples after the cooks, excluding the cook at 140 °C (because of the incomplete dissolution of this sample in Cupriethylenediamine (CuED) even after a holocellulose stage). At a high temperature (165 °C), cellulose was more degraded with a final DPv at a range of around 1750–1800 (the random breakage of β-O-4 glycosidic linkages in cellulose by alkaline hydrolysis at a high temperature strongly affects the DPv). Subsequent secondary peeling reactions also explain the yield decrease (Figure 1A). Good cooking conditions were observed close to 155 °C, where the highest DPv (2240) and the lowest kappa number (21.3) were observed.

As a summary, it was shown that, depending on the temperature, kraft delignification kinetics of poplar chips was partly controlled by mass transfer (diffusion in the wood matrix) and chemical reactions, resulting in an increasing value of activation energy as the temperature decreased. It might be expected that improving chemical species transfer in the chip wall can improve delignification. In the following part, two pretreatments of the poplar chips prior to kraft cooks were assessed: a mechanical pretreatment (MSD) and an enzymatic (xylanase) pretreatment.

### 2.2. Kraft Cooking of Xylanase and MSD-Pretreated Wood Chips

Using xylanase and/or mechanically pretreated chips with a Modular Screw Device (MSD), poplar cooking experiments were carried out at a unique temperature, 145 °C, using the same cooking conditions as before. At this temperature, delignification is rather slow, but the yield is rather high, and the three delignification phases (initial, bulk, and residual) can be observed. In the previous results, it was shown that pulp quality was lower compared to the one at 155 °C as fibers were less separated. However, it was expected to better highlight differences in cooking behavior, possibly levelled out at higher temperatures (although industrial cooks are generally performed at higher temperatures, typically in the range of 150–170 °C [37]). Xylanase impregnation was tested with and without mechanical action, assuming enzyme penetration in the wood would be enhanced by mechanical treatment. The same properties were assessed (yield, kappa number, REA, microscopic observations, and DPv). Results from the small autoclave reactions are presented in Figure 7A–C (the detailed data are available in Supplementary Materials file in Table S1).

The different cooks are denominated as follows:

- A temperature of “145 °C” for non-pretreated wood chips;
- A temperature of “145 °C_Xylanase” for rectangular wood chips impregnated with xylanase;
- A temperature of “145 °C_MSD” for MSD-pretreated wood chips (mechanical treatment);
- A temperature of “145 °C_MSD_Xylanase” for MSD-pretreated wood chips, impregnated with xylanase.

- Pulp yield (Figure 7A):

It is shown that for all cooking conditions, the pretreatment causes faster yield decay, especially before 180 min (initial phase), with final values in the same range (53.4 wt.% for rectangular wood chips impregnated with xylanase, 53.6 wt.% for MSD-pretreated wood chips, and 53.9 wt.% for MSD-pretreated wood chips impregnated with xylanase). The latter values are in the range of about 6–7 wt.% below those obtained for non-pretreated chips, above 60 wt.% (the last value of the plateau, 55.4 wt.%, should be considered with some caution).

MSD pretreatment: clearly, the faster yield decay in the early stages of the cook is caused by mass transfer improvement (initial delignification is mass transfer-limited). The diffusion of chemicals and reaction products in the chip wall is accelerated [41]. Possibly, dissolution of wood products in the external liquor (including polysaccharides) occurs, which involve a decrease of the pulp yield. At the end of the cook, more lignin and hemicelluloses are lost.

Xylanase impregnation: in this case also, a faster and continuous yield decrease is observed compared to non-pretreated wood chips, without a clear separation between initial and bulk phases. One possible explanation could be the selective degradation and partial removal of xylans, as previously described by Akgül et al. [22], which indicates that xylanase can penetrate wood chips, to a certain extent, at the fiber microscale but probably not at the nanoscale. This explanation is at the hypothesis stage and has not been evaluated here but opens the door to further analyses of the sugars and black liquor composition after cooking. The yield decreases linearly during bulk delignification, but during the residual phase, a plateau can be observed. It can be hypothesized that this phenomenon occurs due to the gelling behavior of partially degraded xylans in the cell wall because of the enzyme action, increasing viscosity, and hindering mass transfer for reagents and wood reaction products, such as partially degraded hemicelluloses [22]. Similarly, the kappa number also decreases very slowly (Figure 7C). This shows that the diffusion of lignin fragments in the matrix is also hindered, confirming the action of the enzyme on wood product mass transfer in the cell wall. However, no direct evidence for gel formation due to xylans could be measured in our work.

- REA (Figure 7B)

MSD pretreatment: thanks to MSD pretreatment, chemical impregnation is faster at the beginning of the cook. But when a high-temperature plateau is reached, alkali consumption is almost the same as without MSD treatment, showing that the liquor similarly reacted with wood components with or without MSD pretreatment.

Xylanase impregnation: alkali consumption in this case is smaller, and this was also observed for MSD–xylanase wood chips. One hypothesis is that partially degraded xylans, generated by enzymatic pretreatment, present fewer reducing end-groups susceptible to peeling reactions (responsible for the highest consumption of alkali) compared to intact xylan chains, given that xylan stability toward peeling is known to depend on its substitution pattern [42] and that new reducing ends generated during cooks are a primary driver of alkali-consuming peeling reactions [43]. Moreover, mass transfer hindrance likely limits the diffusion of alkali across wood chips but without affecting the total yield, as good as without enzyme impregnation.

In Figure 8, it can be seen that pulp yield and REA are rather well-correlated. Again, it can be seen that the bottom and more to the right yield curve in Figure 8, distinguished from the others, corresponds to the xylanase pretreatment case (without MSD action). This curve shows that the lowest alkali consumption value is obtained at a given yield. Conversely, the worst result is obtained for the non-pretreated wood chips, but in such a case, the lowest correlation coefficient is obtained, showing more irregular cooking behavior. Interestingly, MSD pretreatment with or without enzymes gives an intermediate result. In the xylanase–MSD case, it seems that the “alkali-saving” effect provided by the enzyme is counterbalanced by the better accessibility of chemicals in the wood matrix, provided by the mechanical treatment. This counteracts the mass transfer hindrance due to xylanase action. Accessibility improvement by the mechanical treatment is probably a dominating effect since with or without xylanase, MSD curves are superimposed.

Overall, it can be concluded that MSD treatment provides a rather poor benefit during wood cook, in terms of the alkali vs. yield result, but it improves pulping homogeneity. It was also shown that the lowest alkali consumption value at a given yield is achieved by xylanase treatment alone applied on wood chips.

- Kappa number (KN) (Figure 7C)

When MSD chips are cooked, the KN decreases faster than without pretreatment and quickly reaches a plateau at about KN = 50 after 160 min and a final value of 41.1 at the end of the cook. Bulk delignification is reached more quickly (without “kinetic delay”) because of the lack of mass transfer limitation (jump over the initial phase). This was observed with and without enzyme impregnation. The same effect was observed for MSD–xylanase wood chips, but the final kappa number was slightly higher (46.6 on the final pulp), probably because of mass transfer hindrance by xylanase. This correlates with the yield curve and confirms that extending the cooking time is unnecessary and that the cook could be stopped earlier than at the end of the long plateau applied in our case.

Regarding xylanase-impregnated wood chips, the transition between initial and bulk delignification is as smooth as without impregnation. Here, again, no residual delignification was observed. The final kappa number is higher (49.6), likely due to diffusion hindrance for lignin moieties inside the wood matrix, as already discussed. To a lesser extent, some diffusion limitations also take place for MSD–xylanase wood chips. This hypothesis for the effect of xylanase has been proven by Akgül et al. [22], who showed that xylans precipitate onto cellulose fibers and can prevent lignin removal.

- Fiber structure analysis (Figure 9 and Figure 10)

Figure 10 shows that mechanical pretreatment causes an increased degradation of the fiber structure, resulting in a reduction in fiber length and an increase in the proportion of fines elements. These characteristics are also visible in the microscopic and SEM images of the final pulps, where the effect of mechanical pretreatment is clearly visible (Figure 9A–H). Furthermore, SEM images reveal the fibrillation of the fiber surface; the fibers generally have a less smooth wall structure. MSD pretreatment damaged the fibers by compression and shear forces and modified fiber structure. Xylanase impregnation had an effect on fiber morphology: the fiber length increased but the fines content decreased, which is not visible in the optical or SEM images. Lei et al. [19] confirmed that the action of xylanase alone helps to maintain a high fiber length and reduces fines generation. Xylanase enzymes loosen the fiber structure and reduce the number of random cleavages of the fibers. However, when xylanase treatment is applied together with mechanical pretreatment, it is shown in Figure 10 that the fibers are more fragile and severely damaged. Nevertheless, the optical images and SEM observations reveal the low quality of the fibers due to the presence of non-individualized elements (shives) in these cooking conditions, as we already observed in the first part. It is possible that the temperature of 145 °C might be too low to obtain great fiber separation with good quality.

Finally, it is shown in Figure 11 that the DPv of the final pulps decreases when wood chips are mechanically pretreated (1715 for MSD wood chips vs. 2190 for untreated wood chips). Better reagent accessibility and diffusion in the fiber wall increase the hydroxide ion concentration near the cellulose chains. When MSD–xylanase pretreatment is applied, the DPv is slightly higher (1890), in line with previous results from Akgül et al.’s study [22], reporting that xylanase breaks lignin–carbohydrate bonds and generates deposits onto fibers, which protects them from the effect of a strong alkali concentration. However, the rather low DPv (1730) obtained for the xylanase-impregnated chips does not fit this conclusion well.

These mini-autoclave results are comparable to those of Pandey et al. [34], who investigated a similar combination of mechanical destructuration (using an Impressafiner) and xylanase impregnation prior to kraft pulping *Eucalyptus tereticornis* chips, at an almost similar laboratory scale (four 1 L autoclaves in a rotary digester). In their study, mechanical disintegration alone enabled a 15 °C reduction in cooking temperature while maintaining a comparable kappa number and pulp yield. They attributed this observation to improved cooking liquor penetration into the opened wood structure, an interpretation consistent with the faster bulk-phase kinetics observed here for MSD-pretreated chips. Combining the destructuration of wood chips with xylanase impregnation further reduced the kappa number by 26.9% relative to the untreated control (18.2 vs. 24.9) at an even lower cooking temperature (140 °C), at the cost of a 2-percentage-point decrease in screened pulp yield (47.4 wt.% vs. 49.4 wt.% for destructured-only chips). These results are qualitatively in line with the yield decrease we observe upon xylanase impregnation in the present work (53.4 wt.% vs. ~55 wt.% for untreated chips at 145 °C), possibly ascribed to partial xylan removal in both cases.

However, an important divergence concerns fiber quality. Pandey et al. reported that mechanical disintegration improved fiber separation (via FE-SEM observations) and physical strength with no indication of fiber shortening or fines generation. In contrast with the present study, MSD pretreatment led to a reduction in fiber length and an increase in fines content, together with a significant DPv drop (1715 vs. 2190 for untreated chips), even at the mini-autoclave scale. This divergence reflects fundamental differences between the two mechanical technologies: the Impressafiner relies on rotary compression rolls specifically designed to delaminate the chip structure “without damaging the fibers”, whereas the Modular Screw Device (MSD) used here applies more intense shear and compression forces via a screw-press mechanism, more susceptible to direct fiber degradation while exhibiting greater chemical accessibility. This suggests that the mechanical severity and working principle of the destructuring equipment, and not only the pretreatment concept itself, govern the balance between improved cooking kinetics and fiber integrity loss.

### 2.3. Kraft Batch Cooks in Larger Autoclaves and Analysis of Variance (ANOVA)

- Cooking results

Based on the results obtained from the kraft cooking kinetics in small-scale autoclaves previously described, an attempt of cooking industrial wood chips from the same wood in larger autoclaves (6 L), allowing better liquor circulation, was made. Alkali, sulfidity, and liquor-to-wood ratio were the same as in previous kinetic trials in small autoclaves. The temperature was set at 152 °C with a plateau of 4 h, which is closer to industrial common cooking conditions [37] and also because the DPv was the highest in the previous trials (2242 at 155 °C vs. 2194 at 145 °C). Moreover, in the present case, a temperature of 152 °C seems to correspond to the transition between reaction-controlled and diffusion-controlled delignification kinetics, as can be seen on the Arrhenius curve of Figure 3, previously identified with mini-autoclaves.

An important point regarding the use of much larger-volume autoclaves for batch cooks is that they facilitate liquor circulation and improve homogenization and internal movement of the wood chips, compared to the use of mini-autoclaves during the kinetic study. Specifically, the latter had a fill ratio (solids + liquid) of 90% (*v*/*v*) whereas the large autoclaves had a ratio of 40% (*v*/*v*). The superior agitation achieved in the large autoclaves likely enhanced liquor penetration into the wood and the uniformity of delignification.

This difference of filling ratio and free volume is expected to significantly alter chip bed packing and liquor flow patterns. Laakso [44], using a dedicated chip-bed packing simulator validated for mill-scale measurements, showed that excessive packing density and resulting liquor channeling in continuous kraft digesters lead to a non-uniform cook and degraded fiber quality. While this model specifically addresses continuous digesters, the underlying principle—that digester geometry and filling conditions govern the uniformity of chip-to-liquor contact—is possibly transferable to the batch configuration used here, and offers a complementary explanation, alongside differences in thermal mass and heat-up dynamics between the two autoclave scales, for the divergence observed between mini- and larger-autoclave results.

Cooking results are presented in Table 1.

Both mechanical and mechano-enzymatic pretreatments resulted in higher kappa numbers compared to the reference kraft pulp. At the same time, pulp yields increased for the pretreated pulps, with the greatest increase observed for the mechano-enzymatic pulp. Contrary to earlier observations, the latter exhibited the highest alkali consumption.

These results appear, at first glance, to contradict the observations made for mini-autoclaves. Additionally, significant morphological changes in the fibers were noted: they appear to undergo severe erosion when mechanical treatment is applied (a marked decrease in average fiber length and an increase in the proportion of fines), an effect that is further intensified by the enzymatic pretreatment.

These results demonstrate that, unlike the observations for mini-autoclaves, mechanical and mechano-enzymatic treatments may weaken the wood structure, making it more susceptible to erosion under higher mechanical and hydrodynamic stresses experienced in larger autoclaves with a greater free volume. In the case of mechano-enzymatic treatment, the increased release of xylans into the solution could have also contributed to the higher alkali consumption observed. However, these explanations remain hypothetical, as mechanical and hydrodynamic stresses were not directly measured, and no analysis of black liquor composition was performed to confirm xylan release. Further work, including liquor characterization and controlled agitation studies, would be needed to experimentally verify these mechanisms. In particular, Pandey et al. [34] did not report such an amplification effect, but their study was conducted at a single, comparatively small scale (1-L autoclaves), preventing any assessment of how their pretreatments would behave with bigger autoclaves. Therefore, the present work extends the findings by showing that the balance between improved delignification kinetics and loss of fiber/structural integrity is scale-dependent, at least for the MSD pretreatment used here. Nevertheless, the conditions present in larger-scale autoclaves clearly reveal a stronger impact of MSD and MSD–xylanase pretreatments on fiber integrity than that observed at the laboratory scale.

- ANOVA results

A supplementary analysis of the cooking results in Table 1 was performed using ANOVA (analysis of variance). The aim was to determine whether the result variability of cooks performed under different conditions significantly exceeded the variability of replicate cooks performed under identical conditions. The response variables used were the kappa number, the pulp yield, the REA, the fiber length, and the fines content. Each kind of treatment (untreated chips, MSD-treated chips, MSD–xylanase-treated chips) was replicated three times. Each replicate consisted of the combined contents of two autoclaves for a given condition; thus, a total of 18 cooks were performed. The kappa number represents the average of two repeated measurements.

For each response, the null hypothesis assumed that there were no differences between the treatments. The ANOVA F-statistic was calculated as the ratio of between-treatment variance to within-treatment variance and then compared to the critical F-value at a 95% confidence level. Degrees of freedom were calculated as follows: (*k* − 1) for the treatment effect and (*n* − *k*) for the residual term, where k represents the number of treatments (3) and *n* the total number of measurements (9).

Results for all cooks are listed below in Table 2.

The F-value data at the confidence level of 95% can be compared to the critical F-value calculated from the Fisher law.

From the given results in Table 3, it can be seen that the ANOVA indicates that, given the experimental variability of the dataset, neither of the two pretreatments had a statistically significant impact on pulp yield or residual effective alkali, as the calculated F-value was lower than the critical value from the Fisher tables. In contrast, a significant overall effect of the pretreatment was observed for the fiber length, fines content, and kappa number, suggesting that the residual lignin content varied at least in part of the dataset.

It should be noted that the number of replicates used here (*n* = 3) is comparatively low. Poschner et al. [45], using a fully automated recirculation batch digester, demonstrated that at least seven replicate cooks are required to achieve a ± 1 kappa point precision at 95% confidence versus only four for a ± 0.5 wt.% precision on yield. This observation reflects an intrinsically higher relative variability of kappa number compared to yield (coefficients of variation of 2.21–3.60% vs. 0.70–1.63%, respectively, in their recirculating system). This is consistent with the pattern observed in the present dataset (Table 1), where the coefficient of variation in kappa number (7.9–12.3%) is markedly higher than the coefficient of variation in yield (1.1–7.6%), and likely explains why the ANOVA detected a significant treatment effect for kappa number but not for yield or REA (Table 3). Given that our larger-scale batch digester (rotating oil-bath system) is not equipped with an automatic liquor recirculation system like the one used by Poschner et al., and that only three replicates per condition were performed, the reported variability for yield and REA should be interpreted with caution, and a larger number of replicates would be needed to confirm the absence of effect on these two responses.

## 3. Materials and Methods

### 3.1. Materials

- Wood

Fresh poplar (*Populus deltoides × Populus nigra*) wood was collected in a Technological Institute FCBA wood parcel in Burgundy, France. The trees were 15 years old, and the initial moisture content of the debarked wood was about 50%. Two categories of wood chips were produced from the same trees:

- Conventional industrial screened wood chips. Wood logs were debarked and converted into wood chips using a rotating-knives machine. The wood chips were screened by size with a chip screener (Chips Class, Model 71-01, TMI, New Castle, DE, USA) to collect a fraction between 7 mm in thickness and 5 mm in diameter;
- Calibrated rectangular samples. Wood slices were sampled on the debarked wood log, from which rectangular wood chips were cut so that chip thickness was perfectly orthogonal to fiber orientation (and chip length aligned to fiber orientation). The wood chips were calibrated to a size of 30 mm × 5 mm × 5 mm ± 1 mm.

Chemical composition of poplar was obtained, and the wood was composed of 49.1 wt.% cellulose, 21.1 wt.% hemicellulose, 25.4 wt.% lignin, 3.5 wt.% extractives, and 0.9 wt.% ash content. The polysaccharides (cellulose and hemicelluloses) were composed of 51.3 wt.% glucan, 14.4 wt.% xylan, 3.6 wt.% mannan, 0.3 wt.% galactan, and 0.7 wt.% arabinan. The percentages are weight percentages related to the total wood weight.

Both chip categories were stored at −18 °C in sealed plastic bags for a month prior to cooking to limit moisture loss and biological degradation.

- Enzymes

A xylanase solution (ES-2024/HH062702, endo-1,4-β-xylanase, CAS 9025-57-4, EC 232-800-2) was supplied by AB Enzymes GmbH (Darmstadt, Germany) as an aqueous solution containing a 2.2–2.8% range of active enzyme protein produced by *Trichoderma reesei*. The enzyme is active on xylans from various wood sources, with an optimal working temperature of 55 °C and high activity maintained up to pH 8.

- Chemicals

All chemicals used were of analytical grade and supplied by Sigma-Aldrich (Saint Louis, MO, USA). For kraft cooks, a sodium hydroxide (NaOH) solution was prepared at 200 g/L, and a sodium sulfide (Na_2_S) solution was prepared at 83 g/L.

### 3.2. Methods

- Modular Screw Device (MSD)

A Modular Screw Device (MSD) from Andritz (Graz, Germany) consisting of an infinite rotating screw, which leads wood chips into a compression zone, was used to produce mechanically pretreated wood chips (MSD-pretreated wood chips). Initially, wood chips with dimensions between 7 mm in thickness and 5 mm in diameter were steamed for 30 min at 80 °C and placed in the feeder. The resulting wood chips were sieved and classified into three fractions: (i) elements with a thickness lower than 8 mm and a diameter over 7 mm (57.0 wt.%); (ii) elements with a diameter between 7 mm and 3 mm (31.5 wt.%); and (iii) elements with a diameter below 3 mm (11.5 wt.%). For better repeatability of the cooks using these wood chips, the proportions of the screening were respected when filling the vessels prior to different cooks.

- Impregnation of rectangular poplar wood chips by Xylanase

To evaluate the effectiveness of xylanase in delignification, rectangular poplar wood chips were subjected to enzyme impregnation. The chips were first immersed in water and then placed in a vacuum chamber overnight at room temperature to ensure complete saturation of the wood pores. After impregnation, the chips were weighed to determine the amount of water absorbed. They were then immersed in a 2500 ppm xylanase solution, adjusted to pH 7, and placed in a vacuum chamber for 15 min to improve enzyme penetration. The chips and solution were then placed in a sealed plastic bag and immersed in a water bath at 55 °C for 5 h, with hand stirring every 15 min. Under these pH and temperature conditions, the enzyme exhibited optimal activity (around 100%) as described by the supplier (AB Enzyme GmbH, Darmstadt, Germany). After enzymatic treatment, the chips were rinsed under tap water and then immersed in hot water to deactivate the enzyme.

- Batch kraft cooks in mini-autoclaves (used for the kinetic study)

Kraft cooking kinetics were investigated in a mini-autoclave cooking reactor, consisting of 12 independent autoclaves, each with a volume of about 85 mL. Autoclaves are commonly heated in an aluminum block, electrically heated and thermally insulated, alternately rocking at 180°. Each autoclave load consisted of wood and cooking liquor, with a liquor-to-solids (L/S) ratio of 6. For each experiment (individual vessel), 10 g (oven-dried basis) rectangular wood pieces or MSD wood chips, with a predetermined moisture content, were introduced into the autoclave along with a white liquor composed of 9.94 g of a 200 g/L NaOH solution and 12.03 g of an 83 g/L Na_2_S solution, which corresponded to an effective alkali (EA) value of 25%, an active alkali (AA) value of 30%, and a sulfidity (S) value of 34%. The filling volume of the autoclaves was about 90% (*v*/*v*).

The cooking temperature profile comprised two distinct stages: a rapid heat-up period until 100 °C, then a low-temperature plateau (at 100 °C), the same for all cooks, then a rapid heat-up period until an isothermal temperature plateau was reached, between 140 °C and 165 °C; and finally, rapid cooling of the autoclaves in a cold-water bath few seconds after sampling. The different cooking profiles are given in Figure 12.

After cooking, the remaining wood chips and pulp were washed with tap water through a fine sieve (<50 µm) to minimize fiber loss, then disintegrated in 2 L of tap water for 10 min at 3000 rpm using a conventional disintegrator (Noviprofibre, Eybens, France, model N6200_A). The resulting fiber suspension was then filtered through a paper filter (porosity index of 2) in a Büchner funnel. The fibers were air-dried for several days (until a dry matter content of 92 wt.% was achieved) and stored at room temperature. Given the small quantity of material processed, the pulps were not sieved to preserve sufficient materials for subsequent yield, kappa number, morphology, and DPv measurements. This choice may have led to the retention of poorly separated fibers or shives, particularly for cooks performed at 140 °C and 145 °C, where microscopic observations confirmed incomplete fiber individualization.

- Batch kraft cooks in larger autoclaves

Batch kraft cooks were performed using a 6-vessel reactor, each with a 6 L capacity. The reactor was heated by an oil bath and operated under rotation to ensure thorough mixing of the pulp and chemicals during cooking. Two temperature sensors were employed for precise control of the cooking conditions. A batch cooking temperature of 152 °C was selected to enhance delignification efficiency while remaining close to the transition temperature range identified in the kinetic study. The cooking profile was composed of a ramp-up phase of 55 min to reach the plateau temperature of 152 °C. At the end of the cook, the vessels were cooled down to 70 °C in less than 10 min. The temperature evolution during the cook is presented in Figure 13.

For each experiment (individual vessel), 300 g (oven-dried basis) of industrial wood chips or MSD wood chips, with a predetermined moisture content, was introduced into the autoclave along with a white liquor composed of 298.2 g of a 200 g/L NaOH solution and 360.9 g of an 83 g/L Na_2_S solution, which corresponded to an effective alkali (EA) value of 25%, an active alkali (AA) value of 30%, and a sulfidity (S) value of 34% (the liquor preparation conditions were the same as those for the batch cooks in mini-autoclaves). The filling volume of the autoclaves was about 40% (*v*/*v*).

After cooking, the pulp was naturally drained by gravity on a 50 µm mesh-size fabric to avoid fiber loss, followed by washing with clean water until the wash water reached a neutral pH. The obtained pulp was screened to separate cooked and uncooked fibers. A Somerville vibrating screener was used with a plate equipped with 150 µm slots: cooked fibers passed through the slots, and the other fibers were considered uncooked. The total yield (in wt.%) is the quantity of fiber recovered after cooking divided by the wood quantity introduced in the vessel. The accepted fiber yield (in wt.%) is the quantity of accepted fiber recovered after screening divided by the wood quantity introduced in the vessel.

- Kappa number

The kappa number (KN) represents the amount of residual lignin in pulp after delignification. The measurement was carried out according to the ISO 302:2015 standard [46], which is based on the oxidation of residual lignin by potassium permanganate (KMnO_4_) in an acidic medium. Kappa number determinations were performed in duplicate.

- Residual Effective Alkali (REA)

Residual effective alkali in black liquor (BL) was determined by acid–base titration. To eliminate carbonate interference, Na_2_CO_3_ was precipitated by adding 20 mL of a 7.5 wt.% BaCl_2_ solution prior to titration. A 10 mL sample of BL was titrated with a standardized 1 mol.L^−1^ HCl solution, while monitoring by pH measurement until a final pH of 11 was achieved.

- Pulp Yield

Total pulp yield was calculated from a mass balance between the initial wood charge and the amount of unscreened pulp after cooking. The pulp yield expresses the percentage of dry pulp obtained relative to the initial mass of dry wood.

- Fiber morphology analysis

Fiber morphology was analyzed in aqueous suspension using a MorFi analyzer (TechPap, Gières, France). The system ensures controlled dilution, homogenization, and transfer of fibers to the measurement cell. High-resolution images were processed to determine fiber length after skeletonization and reconnection of fiber segments. Elements between 5 µm and 55 µm in length were considered as fines elements during the analysis.

- Viscosity-average degree of polymerization (DPv)

The degree of polymerization (DPv) of cellulose in the pulp was assessed by measuring the TAPPI Viscosity of the pulp dissolved in CuED, according to the TAPPI Standard Method T230 om-94 [47]. The DPv was computed from the TAPPI Viscosity using the following conventional empirical relationship: DPv = [0.75 × (954 log (TAPPI Viscosity) − 325)]^1.105^, where the TAPPI Viscosity is expressed in mPa.s (or centipoise).

Samples containing more than 1 wt.% lignin (kappa number > 15) were subjected to a conventional holocellulose stage [48] prior to measuring the TAPPI Viscosity. Briefly, the holocellulose stage consisted of treating 2 g of oven-dried pulp with NaClO_2_ (34 g.L^−1^) in an acetic acid buffer (pH 4.8 ± 0.2) at 70 °C under reflux for 2 h, followed by thorough water washing and drying at room temperature until a dry content of 92 wt.% was achieved.

- Optical microscopy

The fiber morphology was examined using an optical microscope VHX-7000 (Keyence, Japan). To enhance the contrast and highlight subtle differences between samples, fibers were stained using the Herzberg reagent (a chlorine, zinc, iodine solution prepared according to the ISO 9184-3:1990 standard method [49]) prior to observation, which stained delignified, cellulose-rich fibers in blue violet, while lignified fibers remain yellow to brown, allowing visual identification of incompletely delignified regions within the samples.

- Scanning Electron Microscopy (SEM)

Scanning electron microscopy (SEM) was performed on a JEOL JSM-IT500HR (JEOL, Tokyo, Japan) equipped with a Field Emission Gun. Images were acquired using an accelerating voltage of 5 kV at different magnifications on samples coated with carbon deposits.

## 4. Conclusions

Poplar chips, cut to precise rectangular dimensions, were subjected to kraft cooking in mini-autoclaves at various temperatures to study cooking kinetics, pulp yield, and alkali consumption. Variations in activation energy during the bulk delignification phase demonstrate that mass transfer phenomena (diffusion within the wood matrix) and chemical reactions simultaneously act as rate-limiting steps for this type of wood, which is characterized by a relatively open structure. Furthermore, the impact of mechanical (MSD apparatus) and enzymatic (xylanase) pretreatments on the chips prior to cooking showed that both pretreatments influenced delignification kinetics and pulp yield profiles, although through different mechanisms. The MSD pretreatment accelerated the early stages of delignification, reducing the cooking time required to reach a given kappa number, compared to non-pretreated chips, while xylanase impregnation, applied alone, achieved the lowest alkali consumption for a given yield among all conditions tested. However, these benefits are offset by the persistence of relatively high final kappa numbers and a reduction in pulp yield and DPv (viscosity-average degree of polymerization) for the pretreated wood chips, resulting from increased polysaccharide extraction and improved accessibility of the strong alkali within the wood structure. Combining MSD pretreatment with xylanase treatment did not yield further improvement over either pretreatment applied alone. Overall, these results indicate that, at the process conditions tested here, neither pretreatment provided a net benefit for conventional kraft pulp quality; consistent with the screening scope of this study, the underlying mechanisms proposed to explain these trends are presented as working hypotheses, to be confirmed by dedicated mechanistic investigations.

Larger-scale kraft cooks and statistical analysis (ANOVA) revealed that, when subjected to harsher mechanical and hydrodynamic conditions in autoclaves with larger free volumes, pretreated chips were more fragile and eroded more rapidly—showing reduced fiber lengths and increased fines—than non-pretreated chips. In summary, this work paves the way for further analyses aimed at a more in-depth evaluation of the pulp properties and their potential for use in other applications, such as nanocellulose production, which require a high deconstruction level of the biomass. In this study, the fiber erosion and fines generation already observed here under MSD and MSD–xylanase pretreatment are relevant with the degree of fibrillation desired in screw-extrusion-based lignified nanocellulose fibril (LCNF) production processes [50]. Moreover, many other mechanochemical pretreatment strategies such as extrusion, grinding, high-pressure homogenization, milling, or acid-assisted treatments exist today to assess these fiber modifications prior to nanocellulose production [51]. These findings suggest that the present pretreatment conditions could be further intensified or combined with such approaches to directly target nanocellulose-grade material.

## Figures and Tables

**Figure 1:**
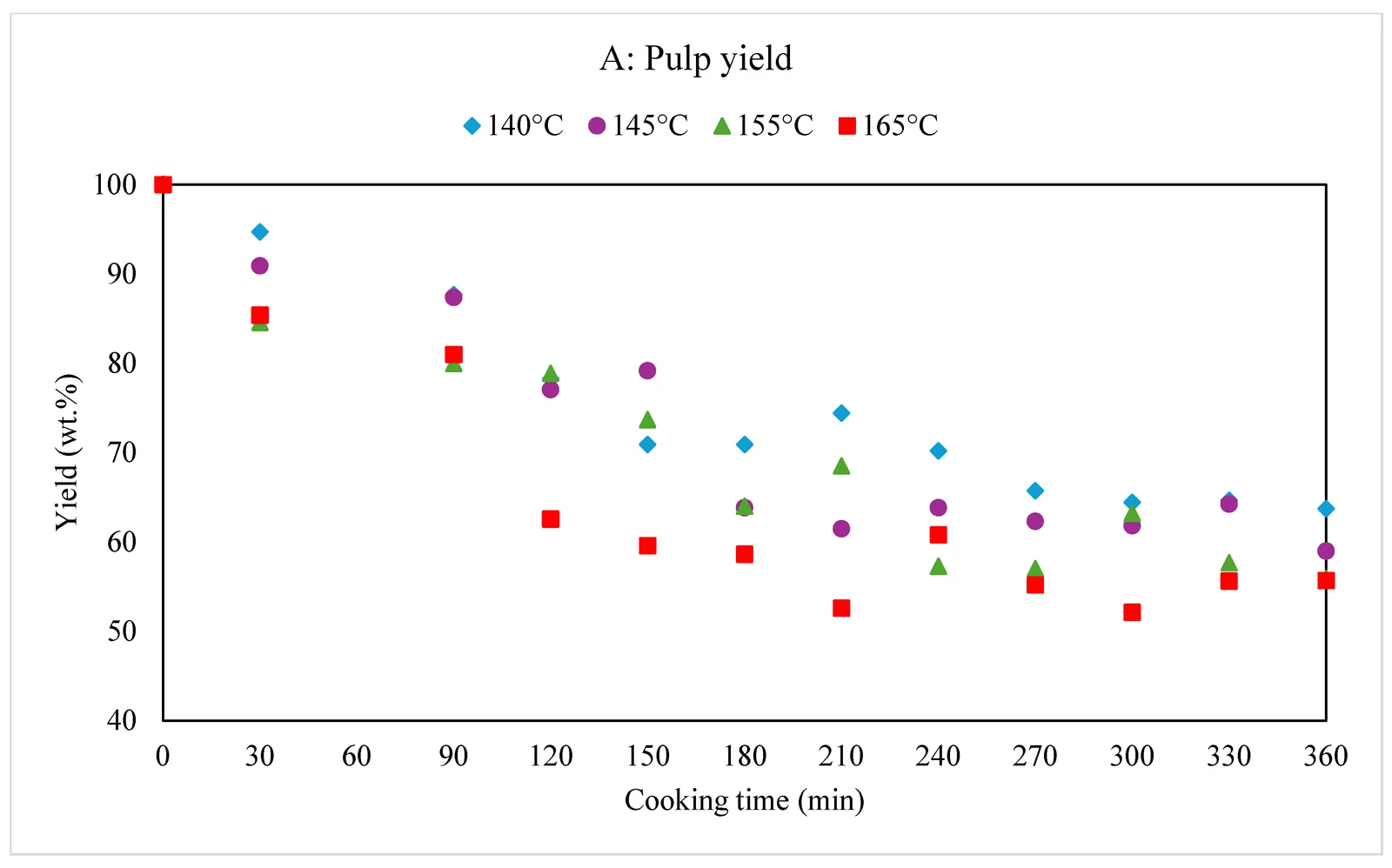

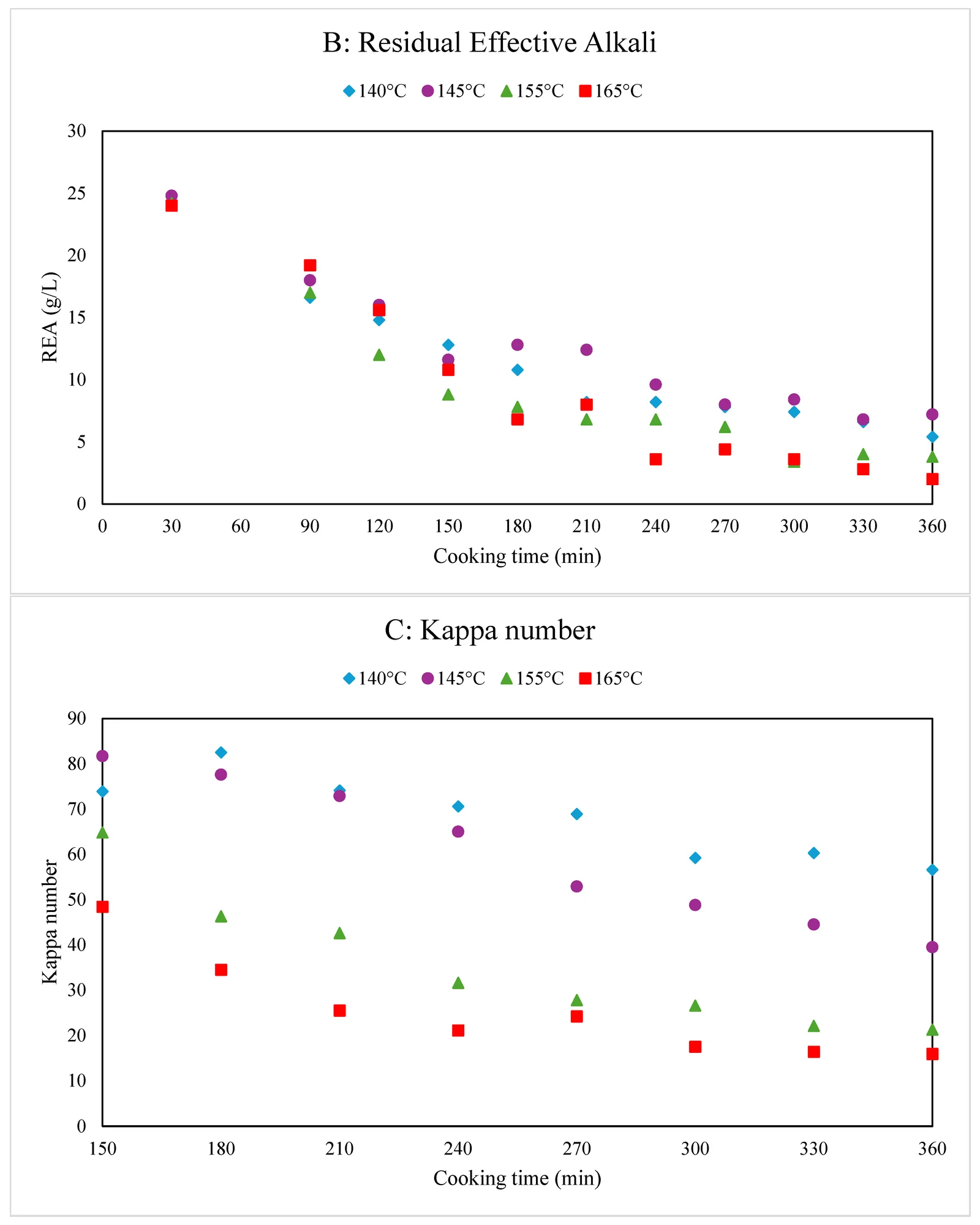
Kraft cooks on rectangular wood chips. Evolution of (**A**) pulp yield; (**B**) residual effective alkali (REA); (**C**) kappa number after 150 min of cooking. Standard deviations of the measured data: ± 1 wt.% for pulp yield, ± 0.5 g/L for REA, and ± 1 kappa number. Small deviations of pulp yield observed on common initial plateau at 100 °C can come from minor fiber losses during pulp washing.

**Figure 2:**
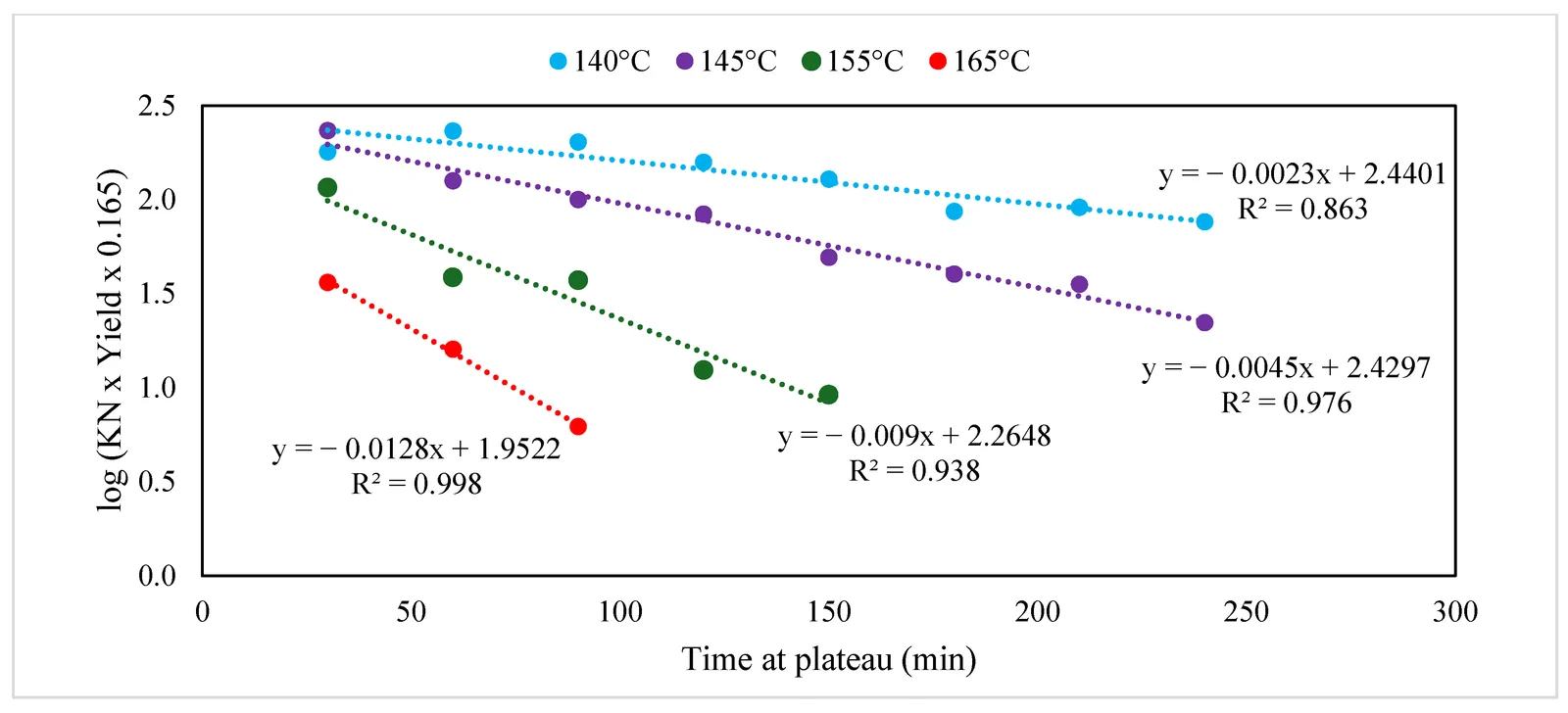
Evolution of log(KN × Yield × 0.165) vs. plateau time for cooks at different temperatures.

**Figure 3:**
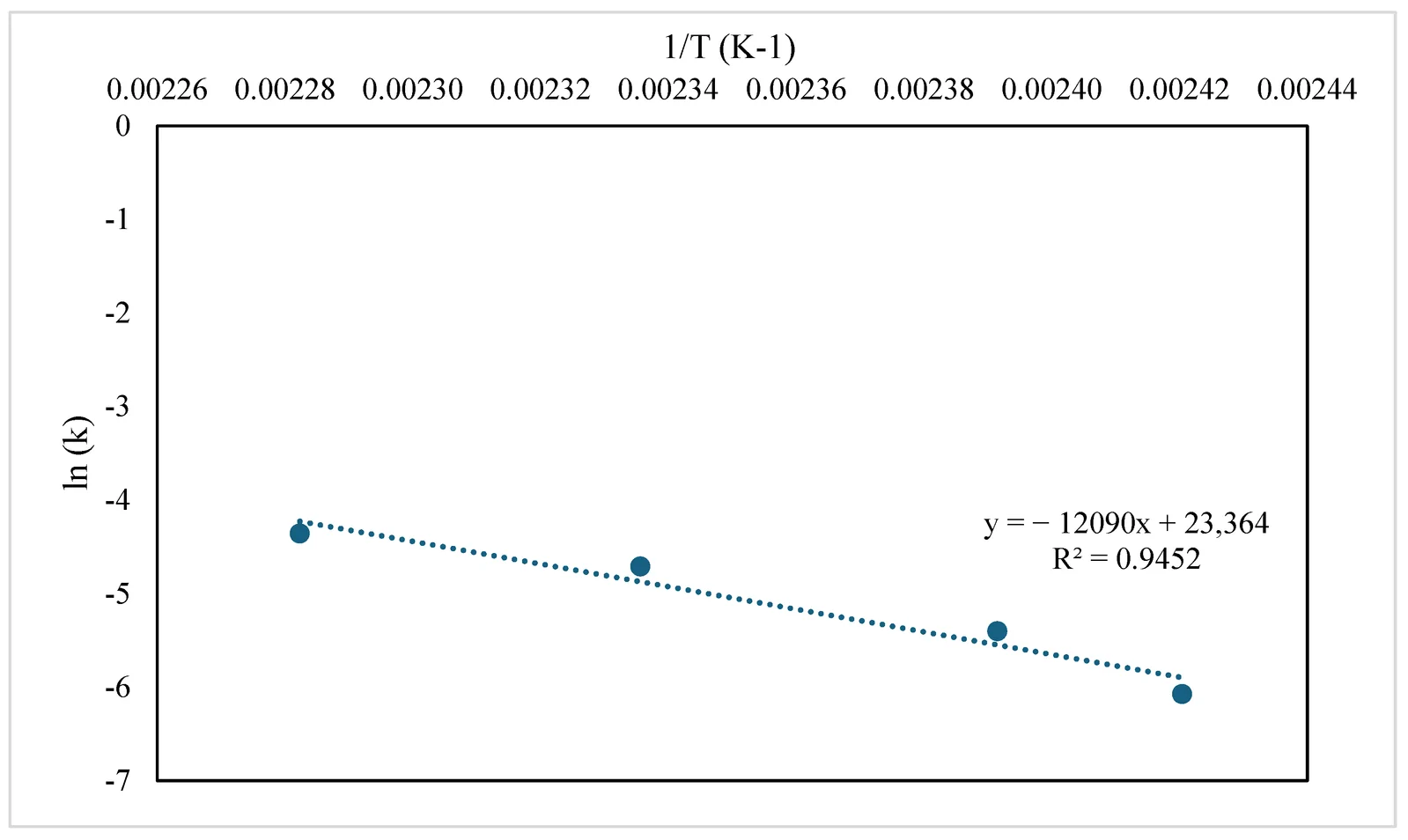
Arrhenius curve representing ln(k) = f(1/T) and its regression straight line.

**Figure 4:**
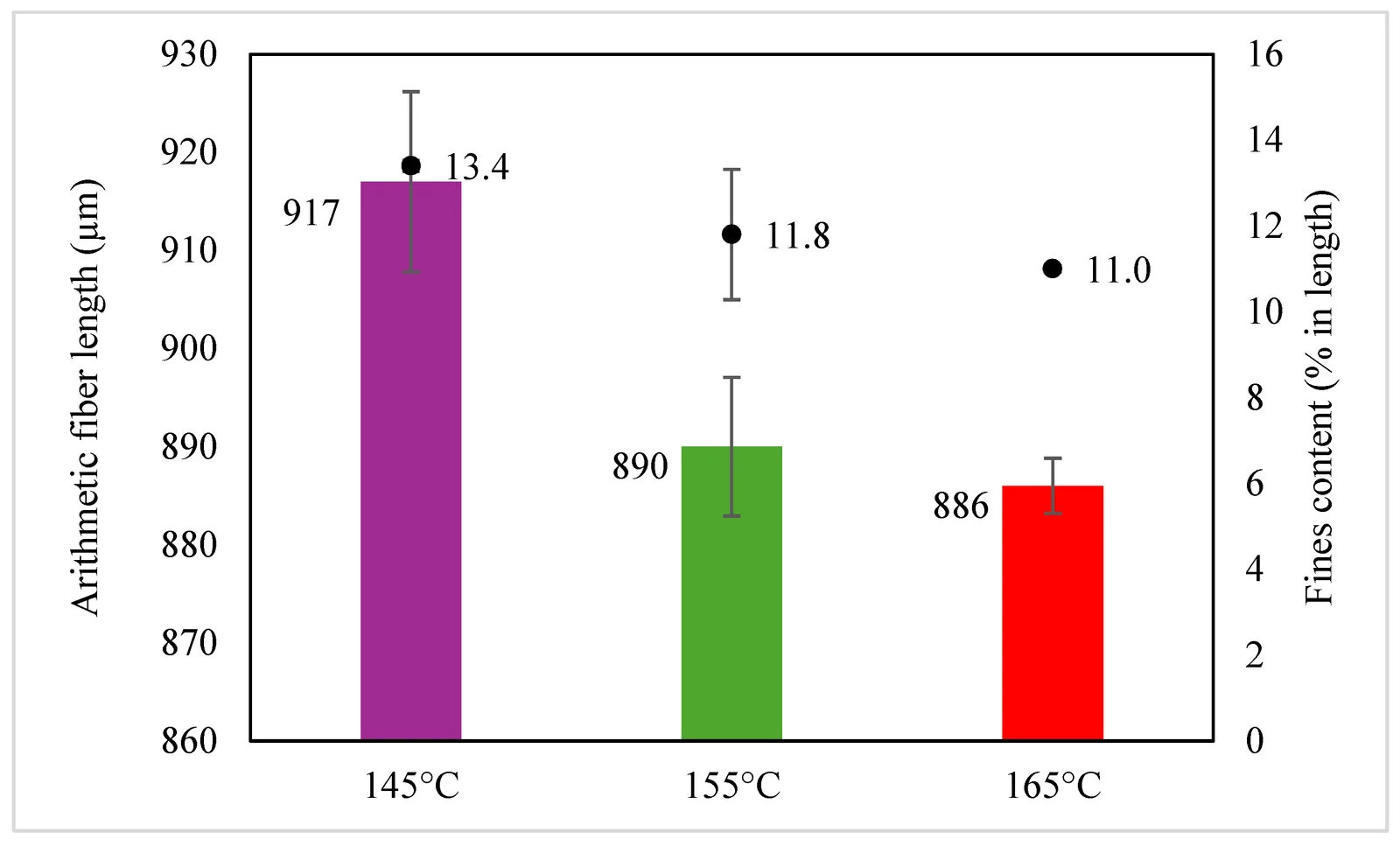
Arithmetic fiber length and fines content in the final pulps obtained for cooks at different temperatures. Kappa numbers of 39.5 at 145 °C, 21.3 at 155 °C, and 15.9 at 165 °C.

**Figure 5:**
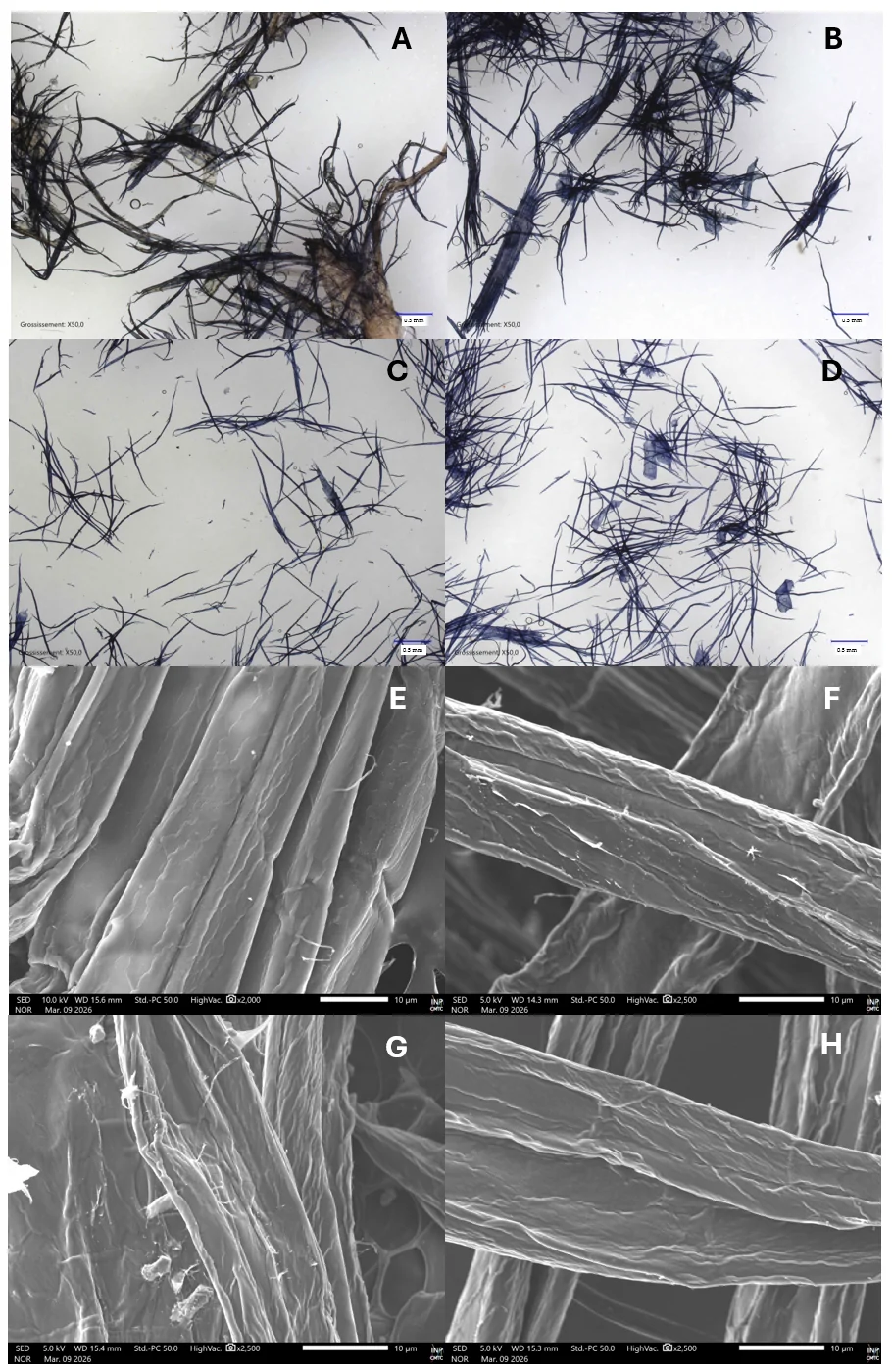
Top: Light microscope images of final fibers at the end of cooks at (**A**) 140 °C, (**B**) 145 °C, (**C**) 155 °C, and (**D**) 165 °C. Bottom: scanning electron microscopy images of final fibers at the end of cooks at (**E**) 140 °C, (**F**) 145 °C, (**G**) 155 °C, and (**H**) 165 °C.

**Figure 6:**
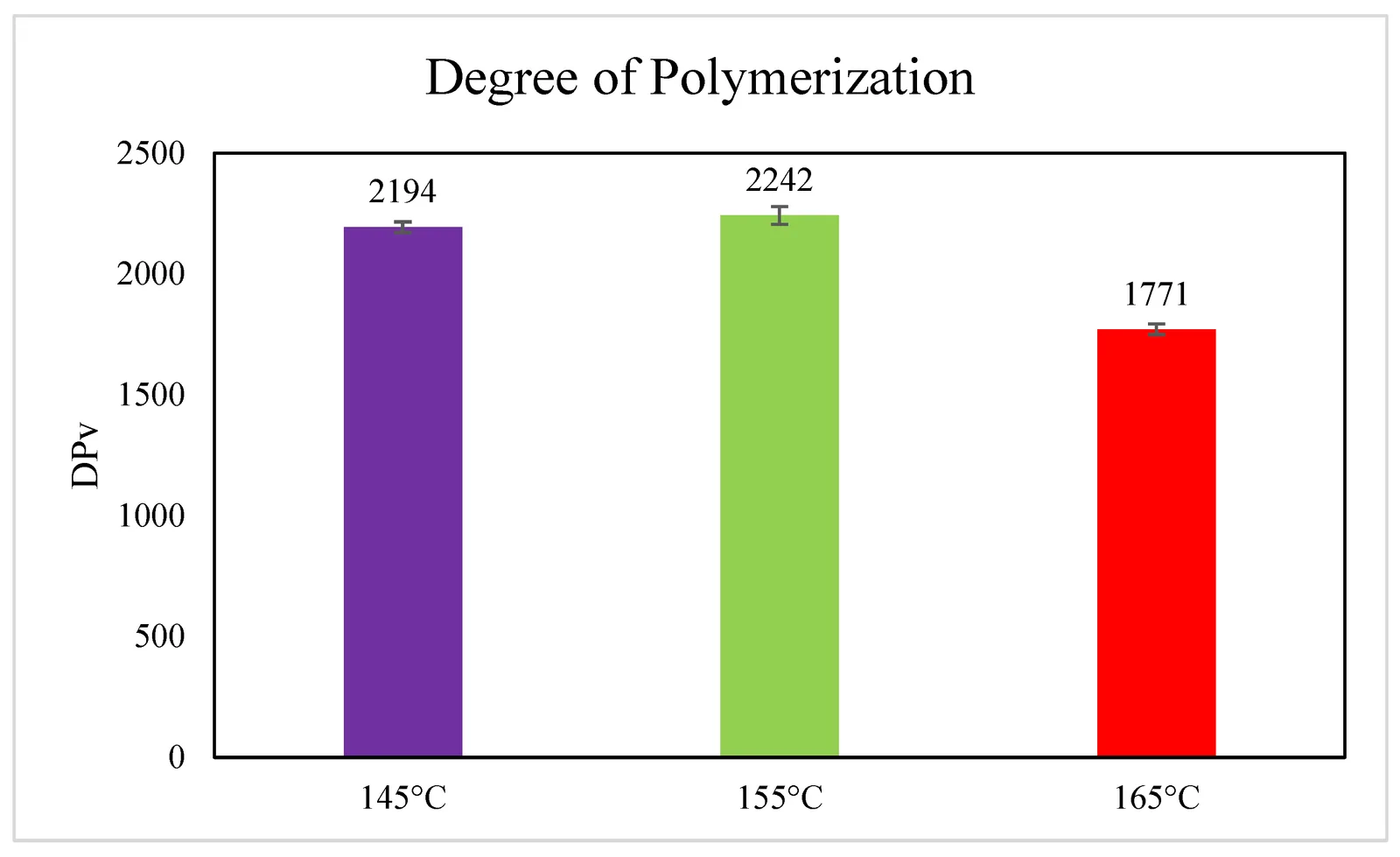
Degree of polymerization of the final pulp of each cook. Kappa number is 39.5 at 145 °C, 21.3 at 155 °C, and 15.9 at 165 °C.

**Figure 7:**
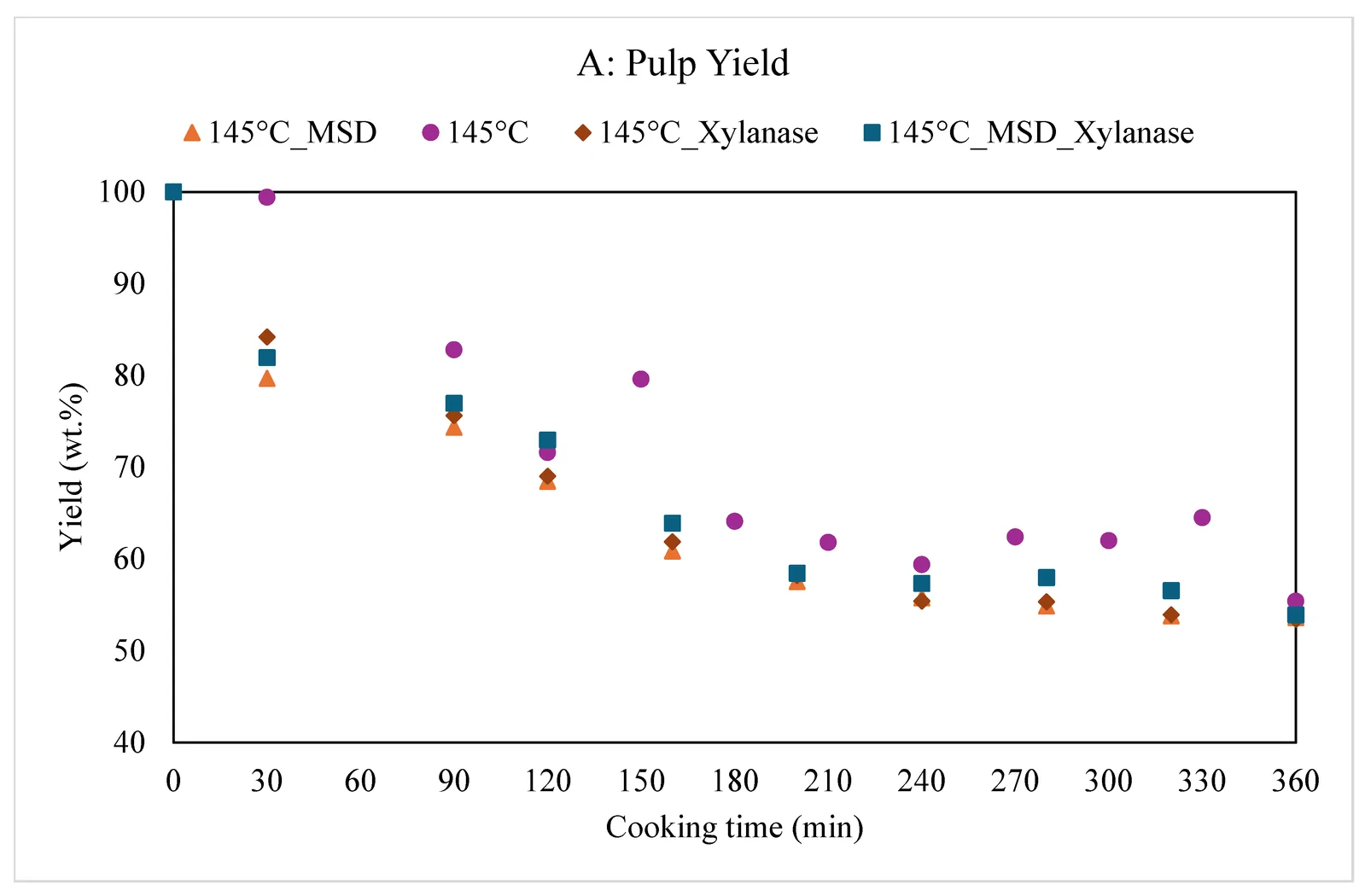

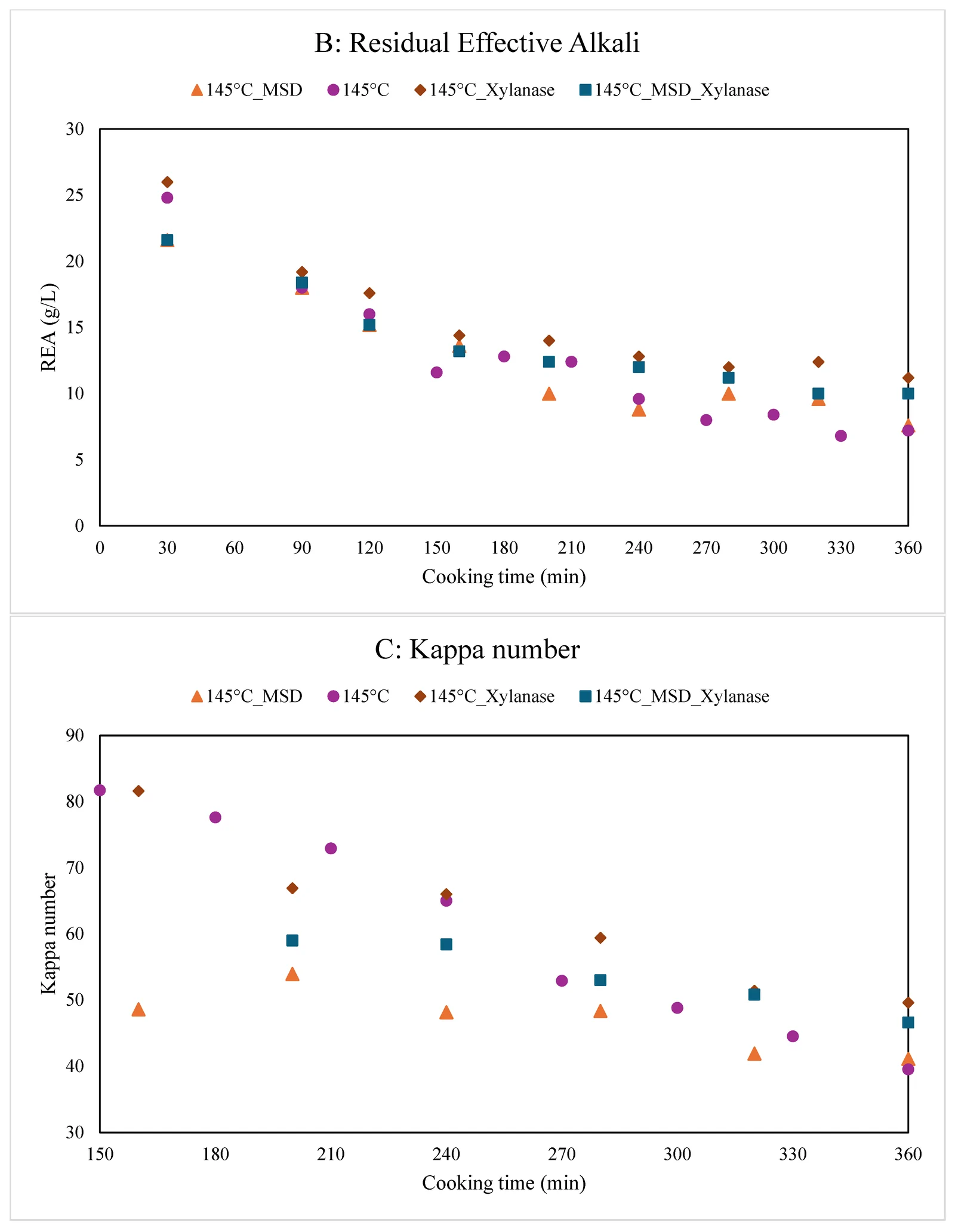
Evolution of (**A**) yield, (**B**) residual effective alkali, and (**C**) kappa number for four cooks on rectangular wood chips and MSD-pretreated wood chips. Standard deviations of the measured data: ±1 wt.% for pulp yield, ±0.5 g/L for REA, and ±1 kappa number.

**Figure 8:**
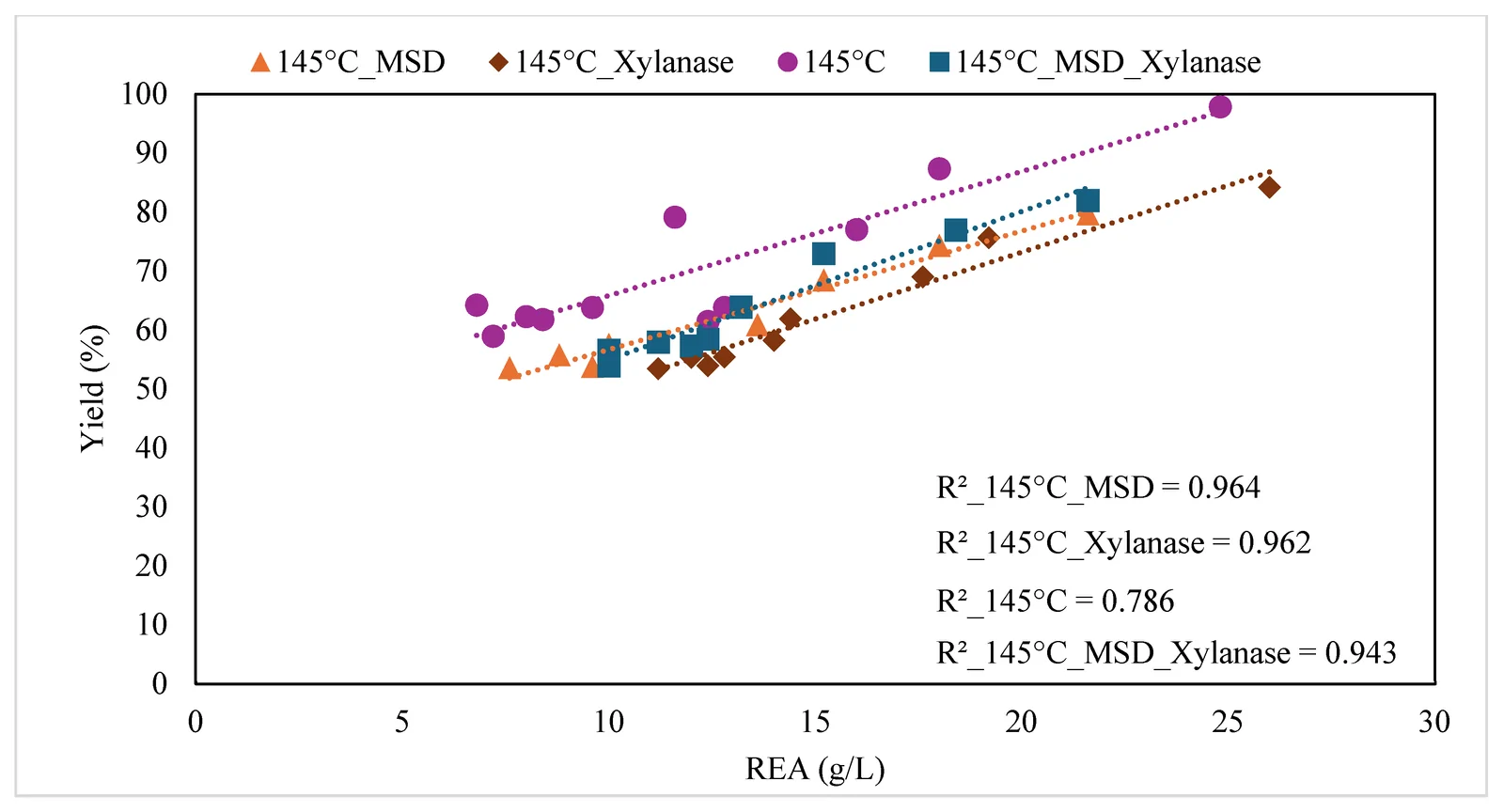
Yield evolution versus residual effective alkali of all wood at 145 °C. Standard deviation for each value is estimated at ±1 wt.% for yield calculation and ±0.5 g/L for REA measurements.

**Figure 9:**
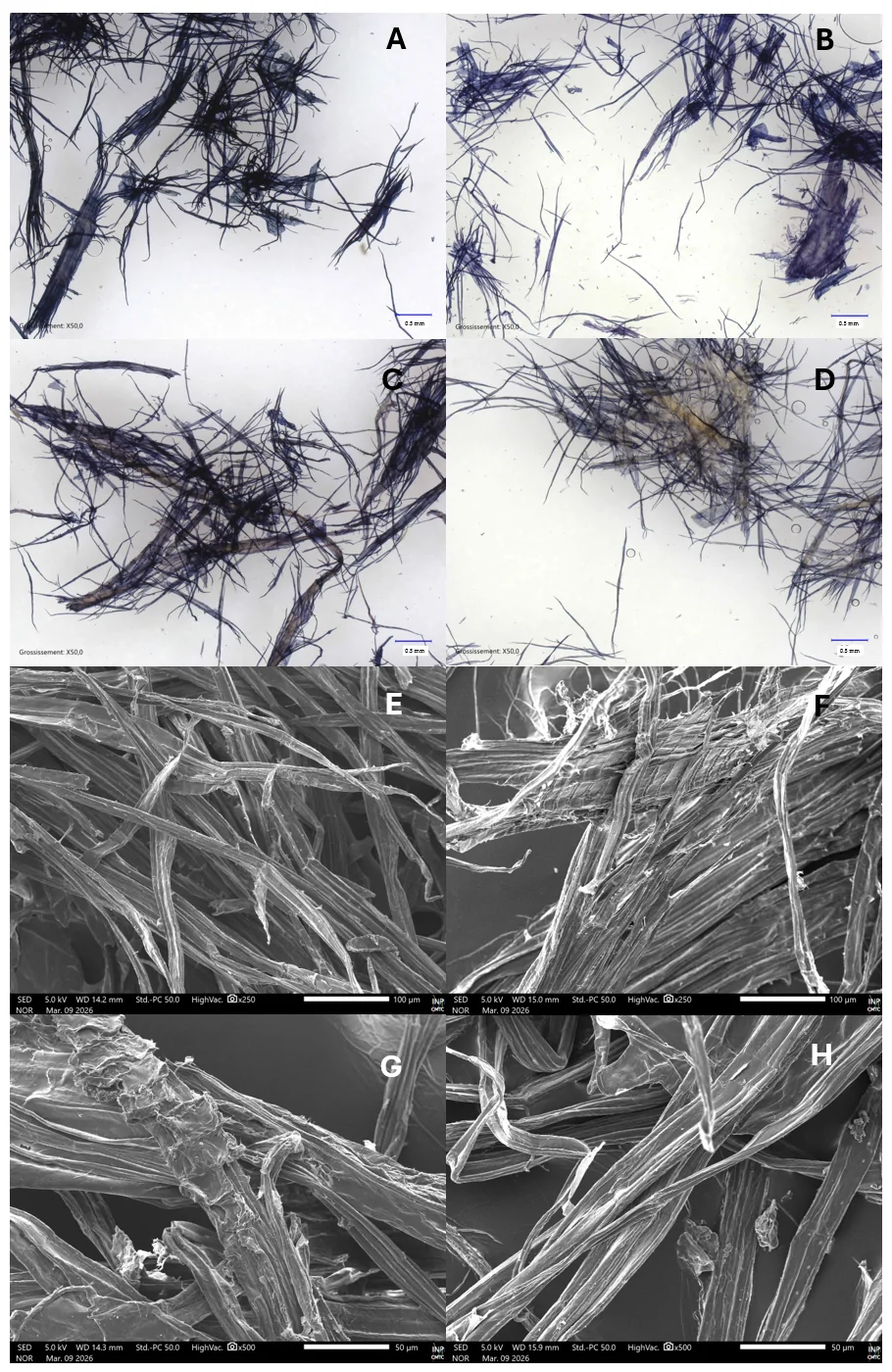
Top: light microscope images of final fibers at the end of the cooks at (**A**) 145 °C, (**B**) 145 °C_MSD, (**C**) 145 °C_Xylanase, and (**D**) 145 °C_MSD_Xylanase. Bottom: scanning electron microscopy images of final fibers at the end of the cooks at (**E**) 145 °C, (**F**) 145 °C_MSD, (**G**) 145 °C_Xylanase, and (**H**) 145 °C_MSD_Xylanase.

**Figure 10:**
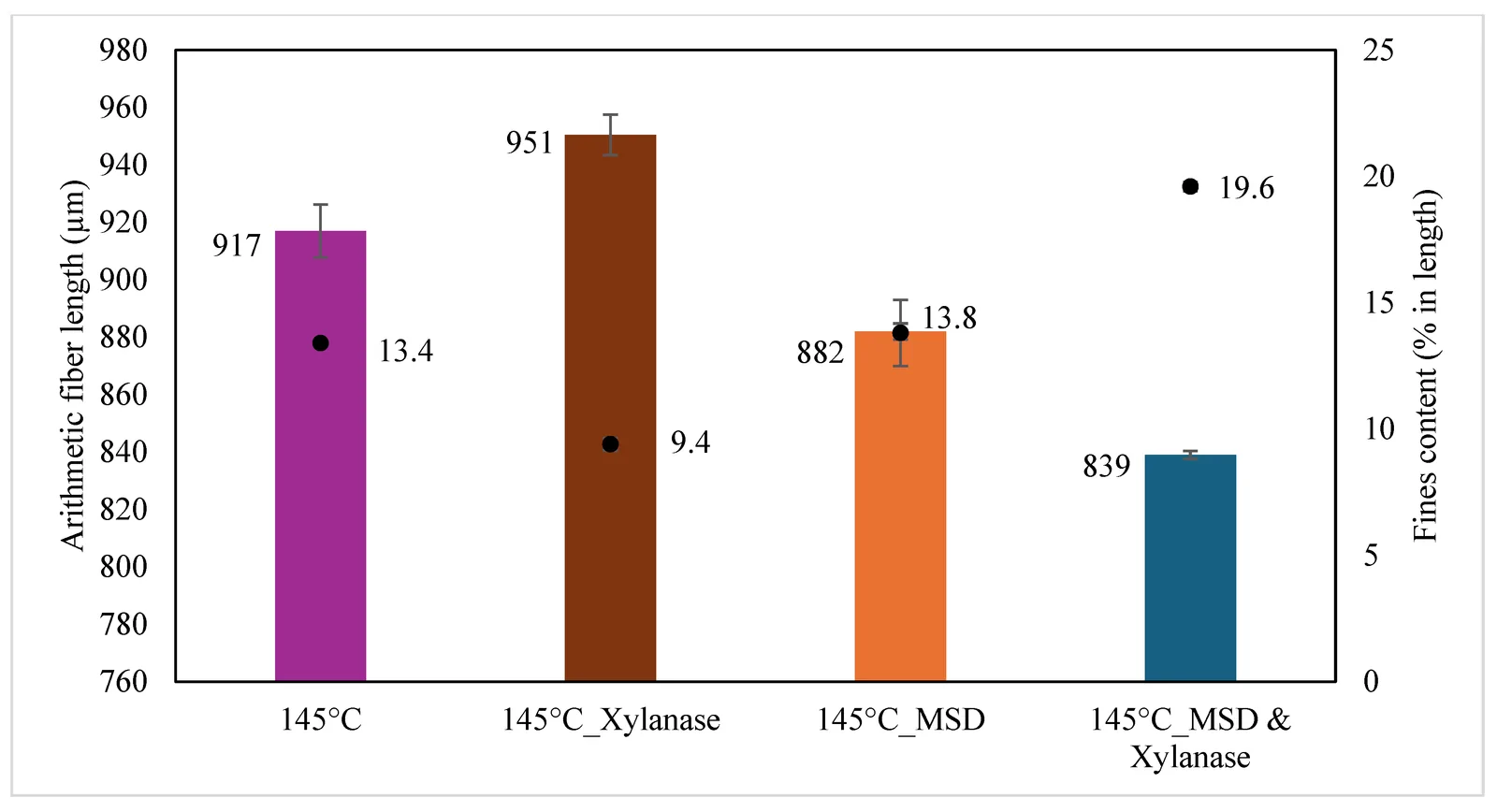
Arithmetic fiber length and fines content of the final pulps obtained for different cooks at 145 °C. Kappa number is 39.5 at 145 °C, 49.6 for 145 °C_Xylanase, 41.1 for 145 °C_MSD, and 46.6 for 145 °C_MSD_Xylanase.

**Figure 11:**
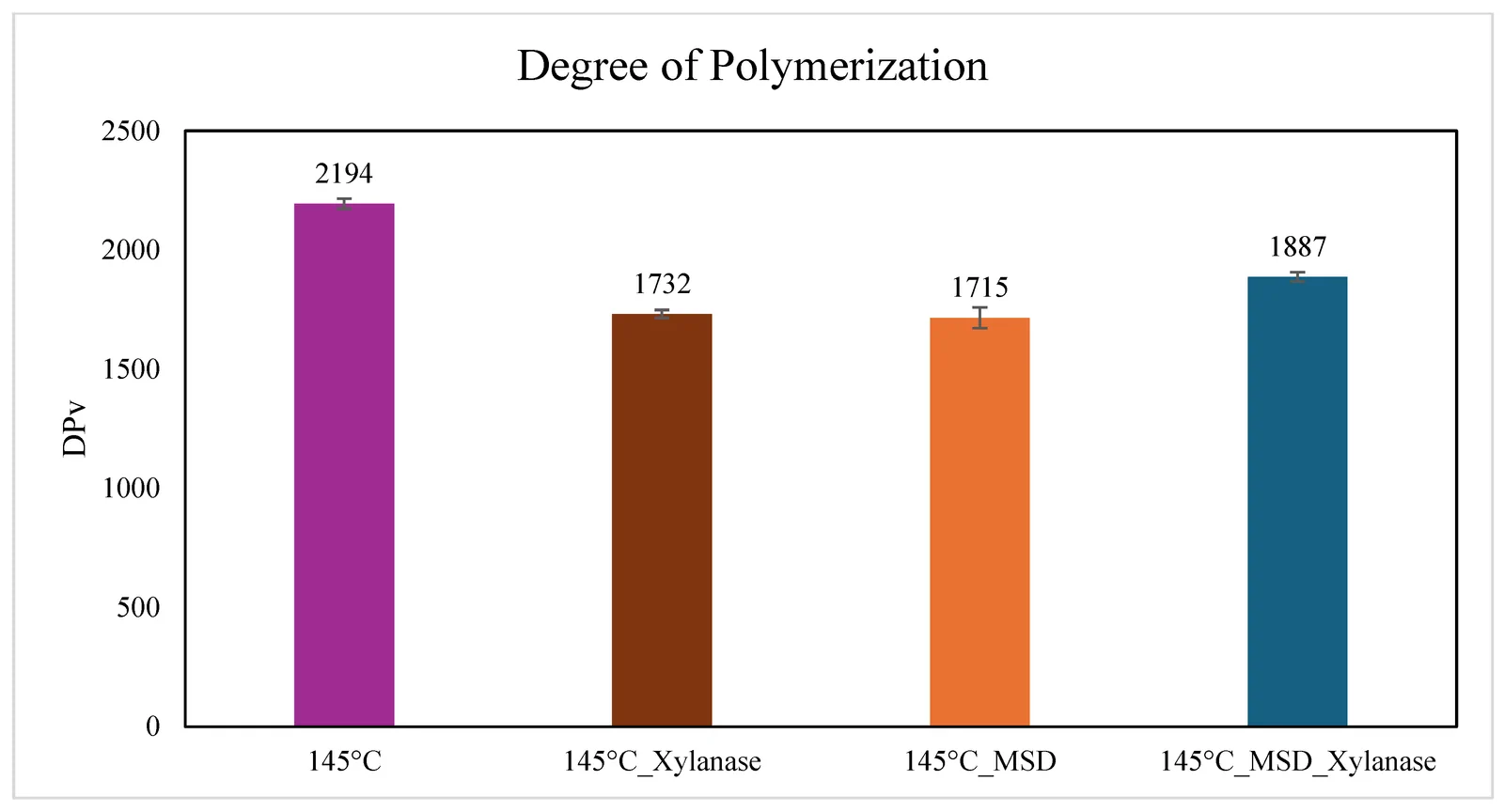
Degree of polymerization of the last vessel of each cook at 145 °C.

**Figure 12:**
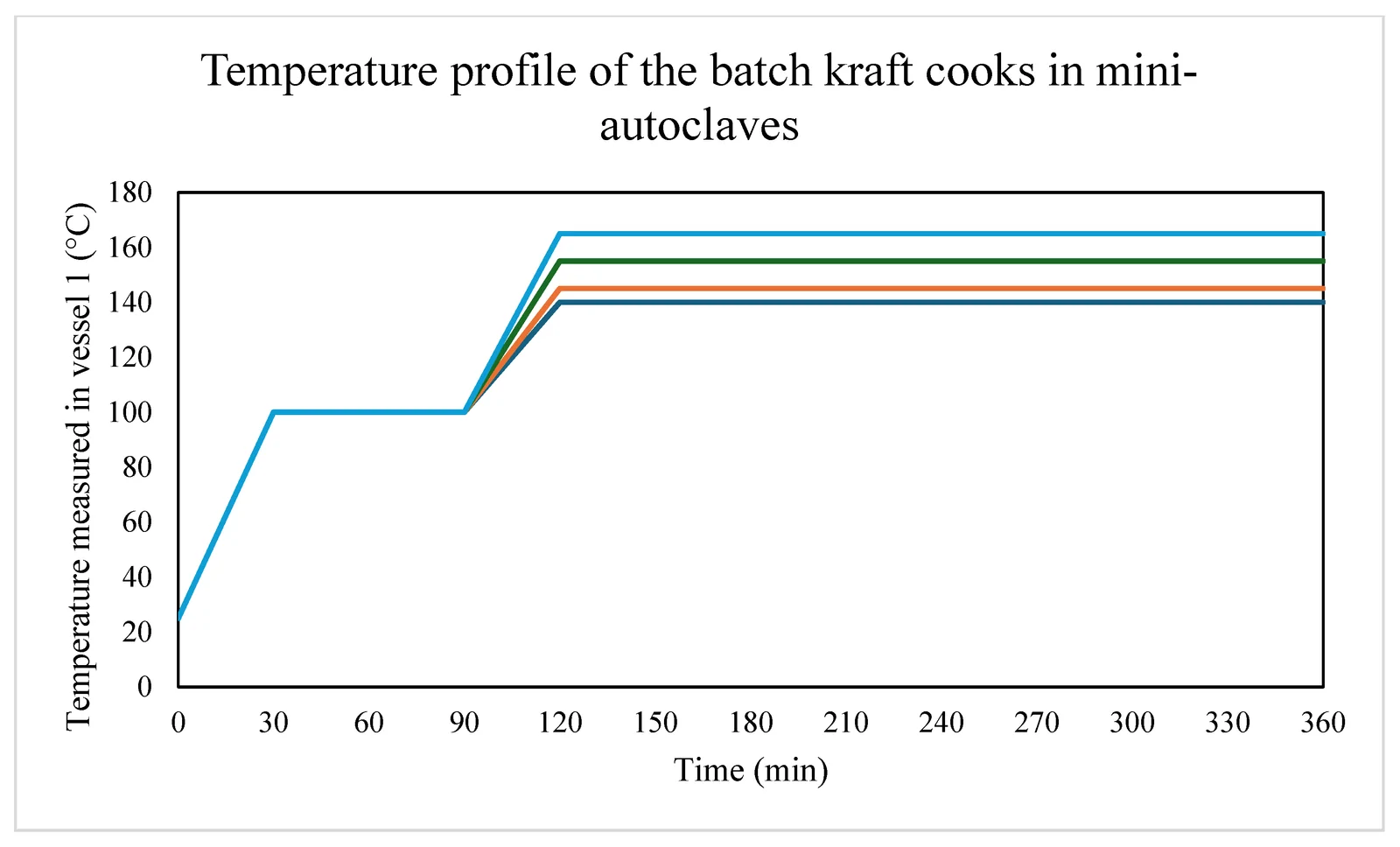
Temperature profile of the batch kraft cooks in mini-autoclaves (used for the kinetic study). The plateau temperatures were 140, 145, 155 or 165 °C.

**Figure 13:**
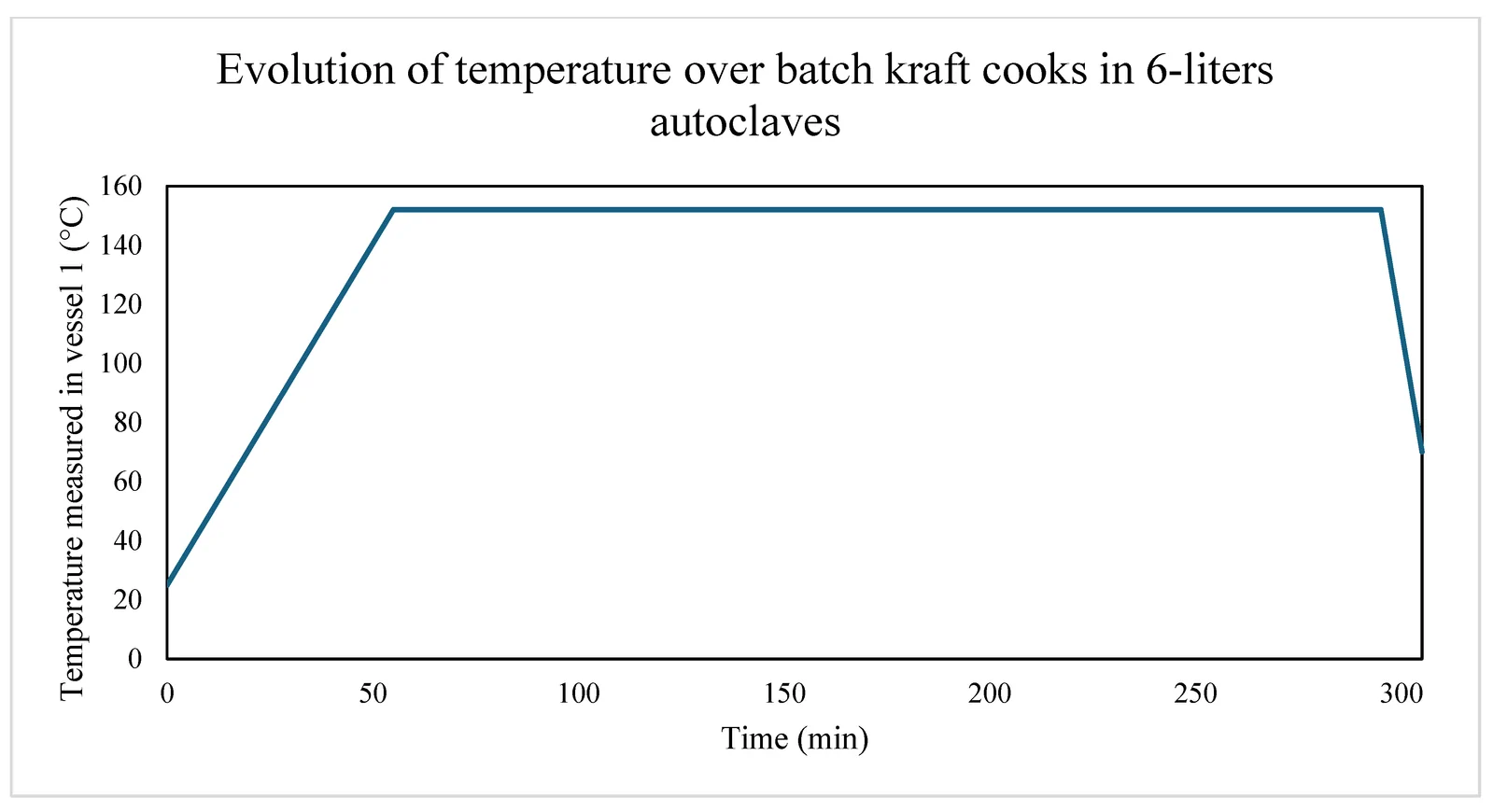
Temperature profile of the batch kraft cooks in larger autoclaves (used for ANOVA).

**Table 1:** Results obtained for batch kraft cooks in three different pretreated conditions.

Reference	Pulp Yield (wt.%)	Accepted Fiber Yield (wt.%)	Kappa Number	REA (g/L)	Fiber Length (µm)	Fines Content (% in Length)
Kraft	51.6 ± 3.9	51.6 ± 3.9	21.9 ± 2.7	8.3 ± 1.5	871 ± 1	20.3 ± 1.3
MSD	52.3 ± 0.7	52.3 ± 0.7	29.2 ± 2.9	8.2 ± 1.2	845 ± 2	26.3 ± 0.1
MSD_Xyl	55.5 ± 0.6	55.5 ± 0.6	30.2 ± 2.4	7.2 ± 0.8	784 ± 2	29.1 ± 0.5

**Table 2:** Dataset used for the ANOVA.

	Kraft	MSD	MSD_Xyl
Kappa 1	18.6	26.8	32.5
Kappa 2	24.5	31.9	27.8
Kappa 3	22.6	28.7	30.4
Yield 1	52.3	51.5	56.2
Yield 2	55.5	52.9	55.2
Yield 3	46.9	52.5	54.8
REA 1	8.8	7.6	8
REA 2	6.4	9.6	6.4
REA 3	9.6	7.4	7.4
Fiber length 1	870	846	785
Fiber length 2	871	843	782
Fiber length 3	870	845	784
Fines content 1	19.98	26.34	28.70
Fines content 2	20.54	26.32	29.46
Fines content 3	20.30	26.33	29.10

**Table 3:** Summary of one-way ANOVA results for batch kraft cook data. F-value corresponds to the value obtained with the ANOVA test. Critical F-value is the tabulated value obtained from the inverse distribution function of F for (1 − a) = 0.95 (https://numiqo.fr/tutorial/f-distribution, accessed on 7 June 2026).

	Kappa	Yield	REA	Fiber Length	Fines Content
F-value	8.68	1.87	0.57	3567.27	818.26
Critical F-value	5.14	5.14	5.14	5.14	5.14

## Data Availability

Data will be made available on request.

## Supplementary Materials

The following supporting information can be downloaded at: https://www.mdpi.com/article/10.3390/molecules31193384/s1, Table S1. Complete experimental raw data of the analysis performed in Section 2.1 for mini-autoclaves cooking results for rectangular wood chips. Table S2. Complete experimental raw data of the analysis performed in Section 2.2 for mini-autoclaves cooking results for Xylanase and MSD-pretreated wood chips.
