# Supplementary material for: Kraft Cooking Kinetics of Poplar Wood Chips Pretreated via a Mechano-Enzymatic Method for Further Nanocellulose Production
Source: Molecules. 2026 Sep 23;31(19):3384. doi: 10.3390/molecules31193384 (PMC13666377; doi:10.3390/molecules31193384)
Supplement: Supplementary file 1 [file molecules-31-03384-s001.zip › molecules-4451962-supplementary.pdf]

Table S1. Complete experimental raw data of the analysis performed in Section 2.1 for mini-autoclaves cooking results for rectangular wood chips.

	Yield (wt.%)			
**Cooking Time (Min)**	**140 °C**	**145 °C**	**155 °C**	**165 °C**
**0**	100	100	100	100
**30**	94.7	90.9	84.6	85.4
**90**	87.7	87.4	80.0	81.0
**120**	78.3	77.1	78.9	62.6
**150**	70.9	79.2	73.7	59.6
**180**	70.9	63.8	64.0	58.6
**210**	74.4	61.5	68.5	52.6
**240**	70.2	63.8	57.3	60.8
**270**	65.7	62.3	57.0	55.2
**300**	64.4	61.8	63.2	52.1
**330**	64.7	64.2	57.7	55.6
**360**	63.7	59.0	55.9	55.7
	**Kappa number**			
**Cooking time (min)**	**140 °C**	**145 °C**	**155 °C**	**165 °C**
**0**	x	x	x	x
**30**	x	x	x	x
**90**	x	x	x	x
**120**	x	x	x	x
**150**	73.9	81.7	64.8	48.4
**180**	82.5	77.6	46.3	34.5
**210**	74.1	72.9	42.6	25.5
**240**	70.6	65	31.6	21.1
**270**	68.9	52.9	27.8	24.2
**300**	59.2	48.8	26.6	17.5
**330**	60.3	44.5	22.1	16.4
**360**	56.6	39.5	21.3	15.9
	**REA (g/L)**			
**Cooking time (min)**	**140 °C**	**145 °C**	**155 °C**	**165 °C**
**0**				
**30**	24	24.8	24.2	24
**90**	16.6	18	17	19.2
**120**	14.8	16	12	15.6
**150**	12.8	11.6	8.8	10.8
**180**	10.8	12.8	7.8	6.8
**210**	8.2	12.4	6.8	8
**240**	8.2	9.6	6.8	3.6
**270**	7.8	8	6.2	4.4
**300**	7.4	8.4	3.4	3.6
**330**	6.6	6.8	4	2.8
**360**	5.4	7.2	3.8	2
	**DPv**			
	**140 °C**	**145 °C**	**155 °C**	**165 °C**
**Mean**	x	2194	2242	1771
**Std Dev**	x	22	37	22
	**Fiber length (μm)**			

	140 °C	145 °C	155 °C	165 °C
**Mean**	x	917	890	886
**Std Dev**	x	7	3	1
		**Fines content (% in length)**		
	**140 °C**	**145 °C**	**155 °C**	**165 °C**
**Mean**	x	13.4	11.8	11
**Std Dev**	x	0.1	1.5	0.1

Table S2. Complete experimental raw data of the analysis performed in Section 2.2 for mini-autoclaves cooking results for Xylanase and MSD-pretreated wood chips.

			Yield (wt.%)	
**Cooking Time (Min)**	**145 °C**	**145 °C_Xylanase**	**145 °C_MSD**	**145 °C_MSD_Xylanase**
**0**	100	100	100	100
**30**	90.9	84.21	79.69	81.95
**90**	87.4	75.64	74.35	76.97
**120**	77.1	69.02	68.48	72.97
**150**	79.2	61.88	60.89	63.91
**180**	63.8	x	x	x
**210**	61.5	58.22	57.56	58.45
**240**	63.8	55.43	55.78	57.34
**270**	62.3	55.35	54.89	57.98
**300**	61.8	x	x	x
**330**	64.2	53.94	53.82	56.57
**360**	59.0	53.44	53.62	53.91
			**Kappa number**	
**Cooking time (min)**	**145 °C**	**145 °C_Xylanase**	**145 °C_MSD**	**145 °C_MSD_Xylanase**
**0**	x	x	x	x
**30**	x	x	x	x
**90**	x	x	x	x
**120**	x	x	x	x
**150**	81.7	81.6	48.6	x
**180**	77.6	x	x	x
**210**	72.9	66.9	53.95	59
**240**	65	66	48.15	58.4
**270**	52.9	59.4	48.35	53
**300**	48.8	x	x	x
**330**	44.5	51.4	41.,9	50.8
**360**	39.5	49.6	41.1	46.6
			**REA (g/L)**	
**Cooking time (min)**	**145 °C**	**145 °C_Xylanase**	**145 °C_MSD**	**145 °C_MSD_Xylanase**
**0**				
**30**	24.8	26	21.6	21.6
**90**	18	19.2	18	18.4
**120**	16	17.6	15.2	15.2
**150**	11.6	14.4	13.6	13.2
**180**	12.8	x	x	x
**210**	12.4	14	10	12.4
**240**	9.6	12.8	8.8	12
**270**	8	12	10	11.2
**300**	8.4	x	x	x

**330**	6.8	12.4	9.6	10
**360**	7.2	11.2	7.6	10
		**DPv**		
	**145 °C**	**145 °C_Xylanase**	**145 °C_MSD**	**145 °C_MSD_Xylanase**
**Mean**	2194	1732	1715	1887
**Std Dev**	22	17	44	19
		**Fiber length (μm)**		
	**145 °C**	**145 °C_Xylanase**	**145 °C_MSD**	**145 °C_MSD_Xylanase**
**Mean**	917	951	882	839
**Std Dev**	7	6	14	1
		**Fines content (% in length)**		
	**145 °C**	**145 °C_Xylanase**	**145 °C_MSD**	**145 °C_MSD_Xylanase**
**Mean**	13.4	9.4	13.8	19.6
**Std Dev**	0.1	0.2	1.3	0.2
